# Rugby-Specific Small-Sided Games Training Is an Effective Alternative to Stationary Cycling at Reducing Clinical Risk Factors Associated with the Development of Type 2 Diabetes: A Randomized, Controlled Trial

**DOI:** 10.1371/journal.pone.0127548

**Published:** 2015-06-01

**Authors:** Amy E. Mendham, Rob Duffield, Aaron J. Coutts, Frank Marino, Andriy Boyko, David J. Bishop

**Affiliations:** 1 School of Human Movement Studies, Charles Sturt University, Bathurst, NSW, Australia; 2 Sport and Exercise Discipline Group, UTS: Health, University of Technology Sydney (UTS), Sydney, NSW, Australia; 3 Greenmeadows Medical Centre, Port Macquarie, NSW, Australia; 4 Institute of Sport, Exercise and Active Living (ISEAL), Victoria University, Melbourne, VIC, Australia; Tokyo Institute of Technology, JAPAN

## Abstract

**Introduction:**

The present study investigated whether rugby small-sided games (SSG) could be an effective alternative to continuous stationary cycling (CYC) training at reducing clinical risk factors associated with the development of type 2 diabetes mellitus (T2DM).

**Methods:**

Thirty-three middle-aged (48.6±6.6y), inactive men were randomized into a CYC (n=11), SSG (n=11), or control (CON, n=11) group. Participants trained 3d^.^wk^-1^ for 8 weeks, while control participants maintained normal activity and dietary patterns. Exercise duration was matched between groups, which involved CYC or SSG (four quarters, interspersed with 2-min passive recovery). Both training programs were designed to induce similar internal loads of maximal heart rate (~80-85%HR_max_) and rating of perceived exertion. Pre- and post-intervention testing included dual-energy x-ray absorptiometry scan, graded exercise test, fasting 2h oral glucose tolerance test and resting muscle biopsy. Western blotting was used to assess the content of skeletal muscle proteins associated with mitochondrial biogenesis and glucose regulation.

**Results:**

Both CYC and SSG increased VO_2_ at 80%HR_max_, and reduced glycated haemoglobin, glucose area under the curve (AUC; SSG, -2.3±2.4; CYC -2.2±1.6 mmol^.^L^1^(120min)^1^; p<0.05), and total body fat-mass (SSG -2.6±0.9%; CYC -2.9±1.1%), compared to no change in CON (p<0.05). SSG reduced insulin AUC (-30.4±40.7 µlU^.^mL^1^(120min)^1^; p<0.05) and increased total body fat-free mass (1.1±1.2kg; p<0.05), with no change in CYC or CON (P>0.05). There were no differences within or between conditions for protein content of peroxisome proliferator-activated receptor gamma coactivator-1α, sirtuin-1, p53, glucose transporter-4, protein kinase AKT/PKB, myocyte enhancer factor 2A, mitochondrial transcription factor, nuclear respiratory factor (NRF)-1, NRF-2 or mitochondrial complexes I-V (p>0.05).

**Conclusion:**

Rugby small-sided games is an effective alternative to continuous cycling for improving metabolic risk-factors associated with the prevention of T2DM. Despite such positive adaptations in clinical risk factors, there were no changes in the content of skeletal muscle proteins associated with glucose regulation and mitochondrial biogenesis.

**Trial Registration:**

Australian New Zealand Clinical Trial Registry ACTRN12613000874718

## Introduction

Physical inactivity is strongly associated with the development of chronic diseases such as type 2 diabetes mellitus (T2DM) [1]. Therefore, regular exercise can be a potent primary prevention strategy against chronic disease development that can assist to counter the economic and social repercussions of these diseases [1]. The benefits of exercise for chronic disease prevention have traditionally been associated with systemic adaptations and improvements in clinical risk factors (i.e. fat-mass, lean muscle mass, aerobic capacity, lipid profile) associated with glucose regulation and/or insulin sensitivity [1–3]. In part, these clinical benefits have also been attributed to metabolic and molecular remodeling within skeletal muscle [4].

To date, the majority of research has focused on the health benefits of continuous, aerobic-based exercise training, such as walking, running and cycling [4]. However, prolonged intermittent exercise, such as football-specific small-sided games (SSG) training, has the potential to improve motivation and compliance compared with continuous aerobic exercise [5]. Football-specific (futsal) SSG training has also been associated with positive adaptations on measures shown to influence glucose regulation and insulin sensitivity (i.e. body composition, aerobic capacity, blood pressure, capillary and density fiber type), which are either comparable to, or better than, continuous, aerobic exercise training [6–10]. While this suggests that SSG training should be effective for improving glucose regulation and insulin sensitivity, there are no published data directly examining this hypothesis.

Insulin resistance has also been linked with mitochondrial dysfunction and the reduced content of key regulatory proteins, which have been shown to increase with aerobic or high-intensity, low-volume, sprint interval training (SIT) [11]. Recently, SSG training has been reported to increase several factors known to be associated with increases in the content of skeletal muscle proteins important for mitochondrial biogenesis [4, 12, 13]. These factors include increases in maximal oxygen uptake, citrate synthase activity, number of capillaries per muscle fibre, and muscle fibre area [12]. However, to date, there has been no published research directly investigating the effects of SSG training on the content of skeletal muscle proteins associated with mitochondrial biogenesis and improved glucose metabolism.

It has been proposed that exercise training improves insulin sensitivity and glucose uptake, which can be partly explained by increased content of glucose transporter 4 (GLUT4) and protein kinase AKT/PKB [14, 15]. Exercise training has also been reported to increase the content of peroxisome proliferator-activated receptor gamma coactivator-1α (PGC-1α), which in turn regulates mitochondrial transcription factors, function and overall protein content, with over expression associated with improved enzyme activity, insulin sensitivity and aerobic capacity [16]. Additional proteins associated with PGC-1α and mitochondrial functioning are sirtuin (SIRT)1 and p53 [16]. Increased SIRT1 activity has been implicated in the induction of mitochondrial biogenesis following exercise training [17]. While the acute exercise response of p53 has been reported [16], the response to exercise training in humans is unknown. Since SSG training has been reported to improve aerobic capacity, body composition and skeletal muscle structure, it may also improve the content of proteins associated with mitochondrial biogenesis and cellular glucose uptake, so as to improve glucose tolerance.

Understanding the effects of different types of exercise on skeletal muscle regulatory proteins implicated in glucose control and mitochondrial biogenesis, and how they interact with systemic changes in glucose metabolism, may assist to improve preventative and therapeutic strategies for inactive populations. Therefore, the first aim of this study was to compare the effects of continuous cycling and SSG training on risk factors (body composition, aerobic capacity and strength) and indicators (blood glucose metabolism and regulation) associated with the development of T2DM. The second aim was to investigate changes within and between the respective conditions for the content of skeletal muscle proteins that have been associated with glucose control and mitochondrial biogenesis. It was hypothesized that SSG would be an effective alternative to continuous (cycling) training for inactive, middle-age men, capable of eliciting positive changes in clinical risk-factors and skeletal muscle proteins associated with the prevention of T2DM.

## Materials and Methods

### Participants

**Fig 1:**
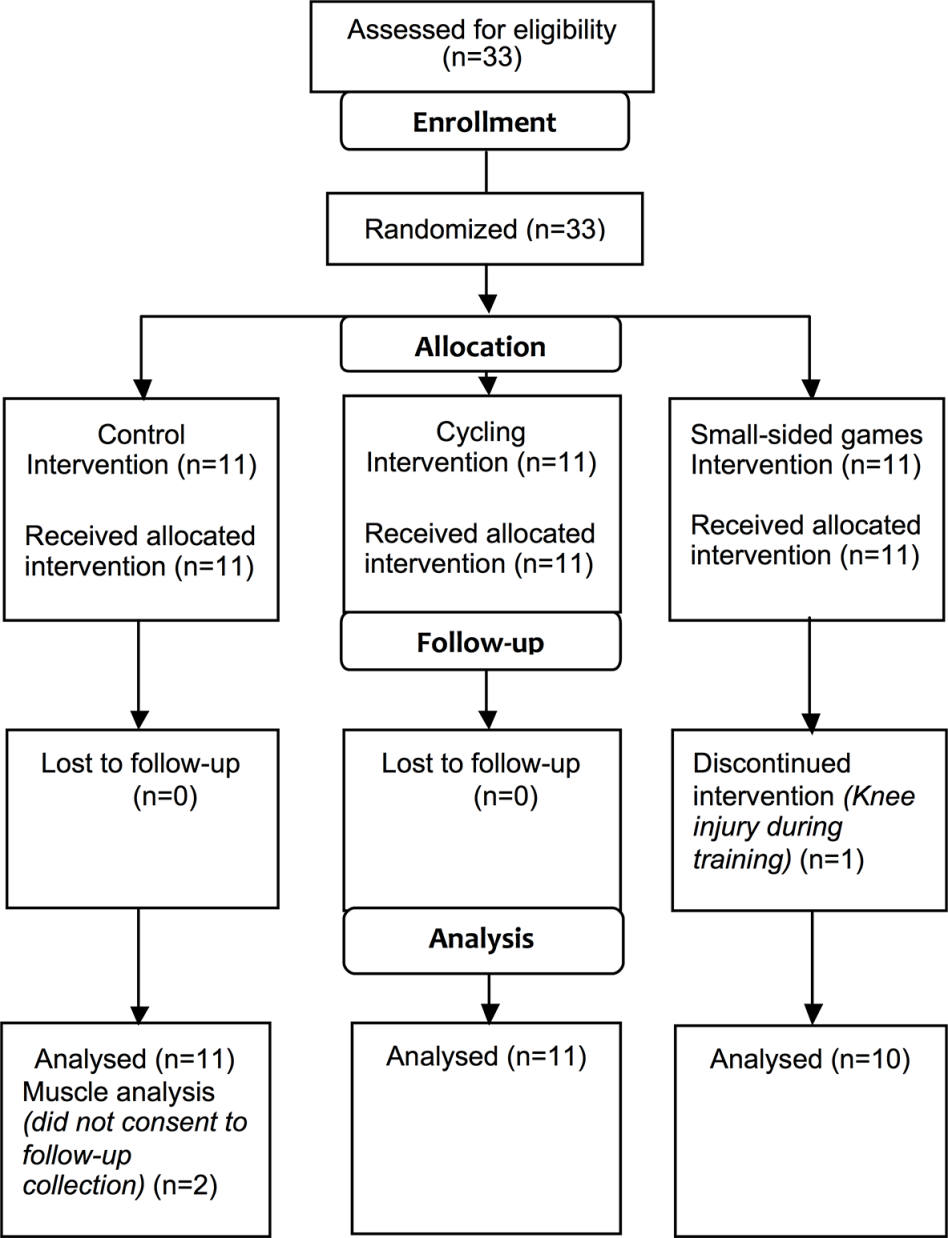
Schematic overview of participant numbers during enrolment, allocation, follow-up and analysis.

Thirty-three men (Table 1; age 48.6 ±6.6 y; stature 176.7 ±5.9 cm; mass 89.8 ±12.3 kg) were recruited and randomly assigned to a stationary cycling (CYC, n = 11), a SSG (n = 11), or a control (CON, n = 11) group (Fig 1).

**Table 1:** Mean ± SD participant characteristics, anthropometry, body composition, aerobic capacity and strength pre and post 8 weeks of control (n = 11), continuous cycling (n = 11) or small-sided games (n = 10).

	Control		Cycling		Small-Sided Games	
Variable	Pre	Post	Pre	Post	Pre	Post
Age *(y)*	49.2 ± 7.0	-	49.5 ± 6.6	-	46.8 ± 6.6	-
Height *(m)*	1.78 ± 0.06	-	1.76 ± 0.04	-	1.76 ± 0.08	-
Body Mass *(kg)*	92.6 ± 11.2	92.8 ± 10.9	90.3 ± 12.3	89.8 ± 12.6	86.3 ± 13.6	86.1 ± 13.4
BMI *(kg* ^.^ *m* ^*2*^ *)*	29.5 ± 3.2	29.4 ± 3.1	29.1 ± 3.8	28.9 ± 3.9	27.6 ± 2.9	27.6 ± 2.9
Waist girth *(cm)*	99.1 ± 8.7	98.5 ± 8.5	96.9 ± 8.6	95.6 ± 9.2	94.9 ± 6.6	92.1 ± 7.5
WHR	0.96 ± 0.04	0.95 ± 0.04	0.95 ± 0.06	0.95 ± 0.07	0.95 ± 0.06	0.94 ± 0.04
**DXA analysis**						
TB-FM *(kg)*	27.1 ± 9.4	27.9 ± 8.6 ^a^	26.7 ± 8.6	25.9 ± 8.4 ^a^ ^,^ ^b^	23.8 ± 6.0	23.1 ± 6.1 ^a^ ^,^ ^b^
TB-FM *(%)*	28.5 ± 7.1	29.5 ± 6.8 ^a^	28.9 ± 6.3	28.1 ± 6.1 ^a^ ^,^ ^b^	27.2 ± 2.9	26.2 ± 3.3 ^a^ ^,^ ^b^
TB-FFM *(kg)*	62.6 ± 6.3	61.8 ± 6.8	60.3 ± 6.3	61.1 ± 6.3	59.3 ± 8.0	60.4 ± 7.8 ^a^ ^,^ ^b^
IA-FM *(kg)*	2.6 ± 1.0	2.7 ± 0.9 ^a^	2.7 ± 1.1	2.6 ± 1.1 ^a^ ^,^ ^b^	2.4 ± 0.7	2.4 ± 0.7 ^b^
**GXT to 80% HR**_**max**_						
VO_2_ *(mL* ^.^ *kg* ^*-1*.^ *min* ^*-1*^ *)*	26.5 ± 5.4	25.7 ± 5.2	24.2 ± 4.1	28.1 ± 3.3 ^a^ ^,^ ^b^	24.7 ± 3.6	28.9 ± 2.9 ^a^ ^,^ ^b^
VO_2_ (L^.^min^-1^)	2.38 ± 0.33	2.30 ± 0.39	2.20 ± 0.60	2.54 ± 0.60 ^a^ ^,^ ^b^	2.14 ± 0.46	2.49 ± 0.43 ^a^ ^,^ ^b^
Workload *(Watts)*	227 ± 33	230 ± 37	207 ± 34	255 ± 31 ^a^ ^,^ ^b^	205 ± 42	243 ± 33 ^a^ ^,^ ^b^
Duration *(min)*	7.5 ± 1.2	7.6 ± 1.4	7.0 ± 1.3	8.6 ± 1.4 ^a^ ^,^ ^b^	6.7 ± 1.5	8.3 ± 1.3 ^a^ ^,^ ^b^
**Strength (3RM)**						
Leg Press *(kg)*	164 ± 49	164 ± 47	182 ± 39	190 ± 36	168 ± 29	193 ± 35 ^a^ ^,^ ^b^ ^,^ ^c^
Chest Press *(kg)*	72 ± 20	71 ± 21	62 ± 11	62 ± 10	62 ± 10	64 ± 11

BMI, Body mass index; WHR, Waist to hip ratio; DXA, Dual-energy x-ray absorptiometry; TB-FM, Total body-fat mass; TB-FFM, Total body-fat free mass; IA-FM, Intra-abdominal fat mass; GXT, Graded exercise test; HR_max_, Maximal heart rate; VO_2_, Oxygen consumption; 3RM, 3 repetitions maximum.

^a^ = Significant within group change (p<0.05)

^b^ = Significant change compared to control group (p<0.05)

^c^ = Significant change compared to cycling group (p<0.05).

A coin toss was used to place the participants into groups of three as they were recruited. After all participants were recruited and groups were formed, block randomisation (Microsoft Office, excel 2010) was used to place each group of three into an allocated condition by the chief investigator. Participant recruitment and follow-up occurred over a 12-week period between October and December 2011. Participants were recruited through verbal communication and newspaper advertisements within a regional Australian community. Based on verbal communication and the self-reporting of activity patterns, participant recruitment ensured the inclusion of participants who were non-smokers, inactive for a minimum of 12 months (i.e. no regular pattern of planned strenuous activity or exercise of >60 min per week), with no clinically diagnosed cardiovascular or metabolic disorders, and taking no prescribed medications. Participants with orthopedic limitations were excluded from the study. Neither the researchers nor participants were blinded to group allocation during pre and post-intervention testing. However, while the training was not blinded, all muscle and blood analyses were blinded.

### Ethics Statement

Prior to participant recruitment and all testing procedures, human ethics clearance was obtained from Charles Sturt University Human Research Ethics Committee (Protocol number: 2011/113; approved September 2011) and participants attended an information and familiarization session where verbal and written informed consent for all testing and training procedures was obtained. This study was registered for inclusion in the Australian New Zealand Clinical Trial Registry and was allocated the clinical trial registration number: ACTRN12613000874718. Clinical trial registration is not an ethical requirement of the University, thus, registration occurred after participant enrollment due to delayed awareness of required clinical registration. The authors confirm that all ongoing and related trials for this intervention are registered. See S1 File (CONSORT checklist), S2 File (protocol as submitted to ethics), S3 File (approved IRB documents) and S4 File (raw data).

### Experimental design

Participants attended two pre- and two post-intervention testing sessions. The first session comprised of resting blood pressure, a sub-maximal graded exercise test (GXT), and a 3-repetition maximum (3RM) chest press and leg press. After a minimum of 72 h recovery from the previous testing session, participants returned for a second session that included anthropometric measurements, a dual-energy x-ray absorptiometry (DEXA) scan, a resting muscle biopsy and a 2-h oral glucose tolerance test (OGTT). The exercise interventions consisted of 8 weeks of SSG (modified rugby) or CYC training. Participants were required to attend at least 90% of all training sessions for their results to be included. Given the different exercise modes, the challenge of matching external training load or metabolic cost is acknowledged. However, in an attempt to match training load between conditions, the respective training programs were designed to elicit similar internal training loads as determined from percent of age-predicted (220-age) maximal heart rate (~80–85% HR_max_) and Rating of Perceived Exertion (RPE) [18].

### Nutritional and physical activity standardization

Prior to the start of all testing sessions participants refrained from any physical activity for 72 h, and the consumption of alcohol and caffeine for 24 h. Participants documented diet and physical activity patterns 24 h prior to pre-intervention testing. This document was photocopied and issued to the participants to ensure diet was replicated for the 24-h period prior to post-intervention testing. Prior to participation in the study, all participants were informed (verbal and written) of the importance of maintaining their normal dietary and physical activity patterns throughout the 8-week training period with lack of compliance resulting in exclusion from the program. Participants were required to maintain food and beverage type and timing of consumption, including cooking preparation and portion size. Physical activity was standardized to ensure all participants in all conditions did not change planned or incidental physical activity during the 8-week intervention.

### Cycling training

Participants performed continuous CYC training (Monark 828E, Monark Exercise AB, Varburg, Sweden) for three supervised sessions per week (details of training load progression are presented in Table 2). To quantify external training load, kilopond (kp), revolutions per minute (RPM) and total distance (km) were recorded at 5-min intervals during each session, with training load and progression manipulated through alternate increases in session duration and resistance (kp). Internal training load was quantified via heart rate (Vantage NV, Polar, Kempele, Finland), which was recorded at 5-min intervals and reported as mean percent of age-predicted (220—age) maximal heart rate (% HR_max_) and session-Rating of Perceived Exertion (session-RPE; Borg 6–20 scale), which was collected 10 min after the conclusion of each training session [19, 20].

**Table 2:** Mean ± SD of session training load over 8-weeks within the continuous cycling and small-sided games conditions.

		Cycling					Small-sided games					
Week	Exercise Duration (min)	Power output (W)	Resistance (Kp)	Cadence (RPM)	Mean Heart rate (%max)	RPE (AU)	Mean Distance (m)	Mean speed (m^.^min^-1^)	Peak Speed (km^.^h^-1^)	Field Size	Mean Heart rate (%max)	RPE (AU)
**1**	40	100 ± 35	1.5 ± 0.3	71.9 ± 7.1	80.5 ± 3.0	11.9 ± 1.0	2563 ± 259	71.5 ± 8.0	20.0 ± 2.9	25m; 40m	86.0 ± 5.2	12.9 ± 1.3
**2**	40	117 ± 38	1.7 ± 0.3	73.7 ± 8.5	83.9 ± 4.2	12.1 ± 1.3	2642 ± 361	73.4 ± 10.1	18.6 ± 2.6	25m; 40m	85.8 ± 4.5	12.2 ± 1.6
**3**	45	121 ± 44	1.9 ± 0.3	76.0 ± 6.4	85.4 ± 4.7	12.3 ± 0.9	2856 ± 298	71.3 ± 7.5	20.0 ± 3.1	25m; 40m	86.5 ± 5.3	12.0 ± 1.2
**4**	45	132 ± 41	2.2 ± 0.3	73.7 ± 5.4	84.9 ± 5.2	12.6 ± 0.8	3014 ± 275	75.1 ± 6.8	21.2 ± 2.8	35m; 50m	85.3 ± 4.1	12.1 ± 1.3
**5**	45	148 ± 33	2.2 ± 0.2	72.3 ± 6.7	85.6 ± 5.2	12.8 ± 0.8	3117 ± 320	77.8 ± 8.0	20.2 ± 3.5	35m; 50m	83.3 ± 5.1	12.3 ± 1.0
**6**	50	146 ± 33	2.2 ± 0.3	70.1 ± 5.6	86.0 ± 4.5	12.8 ± 0.9	3541 ± 369	78.8 ± 8.3	20.9 ± 3.1	35m; 50m	85.8 ± 5.9	12.7 ± 1.0
**7**	50	161 ± 17	2.4 ± 0.2	69.3 ± 5.2	86.4 ± 5.8	12.8 ± 0.8	3673 ± 293	82.0 ± 5.9	20.3 ± 3.4	40m; 60m	84.2 ± 4.5	12.2 ± 0.9
**8**	50	165 ± 19	2.4 ± 0.2	70.1 ± 6.2	84.2 ± 6.4	12.4 ± 0.7	3371 ± 203	80.1 ± 7.5	20.0 ± 3.2	40m; 60m	84.7 ± 5.1	12.5 ± 1.1
**Session Mean ± SD**		136 ± 14	2.0 ± 0.3	72.1 ± 2.6	84.6 ± 2.1	12.5 ± 0.4	3090 ± 421	76.2 ± 5.2	20.1 ± 1.1	33m; 49m	85.2 ± 1.5	12.4 ± 0.4

No significant differences were evident between conditions for heart rate or RPE.

### Small-sided games training

The SSG group performed three supervised sessions per week of modified rugby league. The modified rugby session was played under touch football rules. The rules allowed each team six ‘plays’ while in possession of the ball; each play required players to pass the ball backwards to an ‘on side’ team member with the aim to score at opposing ends of the field. Defending players were required to touch their opponent with one hand. Following a successful touch, game play would restart with a ‘play the ball’, at this time requiring the line of defending players to be 5 m from the position of each ‘play the ball’ [21]. Each training session was comprised of four quarters, interspersed by 2-min passive recovery periods. See Table 2 for details of exercise duration, progression and training load. Notably, total session duration for SSG training incorporates exercise duration and 3 x 2 min passive recovery periods, hence total session durations were 46 min (weeks 1–2), 51 min (weeks 3–5) and 56 min (weeks 6–8). To quantify external training load, a Global Positioning Satellite (GPS) device (SPI-Pro, 1 Hz, GPSports, Canberra, ACT, Australia) was worn in a customized harness between the scapulae to quantify total distance (m), mean speed (m^.^min^-1^) and peak speed (km^.^h^-1^) of each training session. During the 8 weeks, training load progressively increased via manipulation of session duration and field size, including consistent game rules, verbal feedback and player numbers at 5 v 5 or 6 v 6 (depending on participant availability). Heart rate was recorded during, and session-RPE was obtained at the conclusion of all training sessions, to determine internal training load [22].

### Control condition

The CON group completed all pre- and post-intervention testing sessions and was required to continue their normal diet and physical activity patterns over the 8-week intervention period. Participants were provided with a diet and physical activity journal to document any changes. Prior to post-intervention testing each journal was reviewed by the chief investigator to ensure individual conformity to the control condition.

### Measures

#### Anthropometry and DEXA

All testing procedures were conducted at a standardized time between 0600 and 0900 h. Anthropometric measures included stature (Stadiometer, CSU, Bathurst, Australia), body mass on calibrated scales (HW 150 K; A&D, Bradford, MA, USA), waist and hip girths (EC P3 steel tape Sydney, Australia) using standard techniques [23]. These measures were used to calculate body mass index (BMI) and the waist-to-hip ratio (WHR) [23]. Manual blood pressure was obtained with an aneroid sphygmomanometer and cuff (Welch-Allyn, Arden, North Carolina, USA) and expressed as the mean of three measurements after the participant had been seated for five minutes. A supine, whole-body DEXA scan (XR800, Norland, Cooper Surgical Company, USA) was conducted with the scanning resolution set at 6.5 x 13.0 mm, and the scanning speed set at 130 mm^.^s^-1^. Scans were analyzed (Illuminatus DEXA, ver. 4.2.0, USA) for total body fat-mass (TB-FM), total body fat-free mass (TB-FFM) and intra-abdominal fat-mass (IA-FM). Analysis of intra-abdominal fat-mass (IA-FM) was performed with the creation of a 10 cm region of interest standardized across all participants according to previously outlined procedures, with a reported coefficient of variation for fat-mass of 2.6% [24].

#### Graded exercise test

A GXT determined sub-maximal oxygen consumption (VO_2_) and power output at 80% HR_max_ [25]. The GXT was performed on an electronically-braked cycle ergometer (Excalibur Sport, LODE BV, Groningen, The Netherlands), commencing at 25 W and increasing by 25 W every min. Heart rate was recorded each minute throughout the protocol, and participants exercised until attainment of 80% HR_max_. Pulmonary gas exchange was measured by determining O_2_ and CO_2_ concentrations and ventilation to calculate VO_2_ using a metabolic gas analysis system (Parvo Medics, True2400, East Sandy, UT, USA). The system was calibrated according to the manufacturer’s instructions. This involved the pneumotachometer calibration using a 3 litre syringe. The gas analyzers were calibrated using a two-point fully automated process involving room air and gas calibration for fractional gas concentration with a gravimetric gas mixture of known concentrations (CO_2_, 4.1 (0.1)%; O_2_, 15.7 (0.2)%).

#### Maximal strength testing

Following a 30-min recovery period from the GXT, participants completed a 3RM test. Testing procedures determined upper- and lower-body strength via seated chest press and leg press (Panatta Sport, Apiro, Italy), respectively. Participants attempted ascending resistances, separated by a 3-min recovery period, until the determination of upper- and lower-body 3RM. Individual seating positions of head rest, back rest and seat height were recorded and replicated during post-intervention testing.

#### Oral glucose tolerance test

Following an overnight fast (10–12 h), participants presented to the laboratory for a 2-h oral glucose tolerance test (OGTT). Participants were cannulated in the medial cubital vein for venous blood sampling during the OGTT. Following the collection of a fasting venous blood sample, each participant ingested a standardized 75 g glucose solution within a 5-min period and remained rested for the remaining blood collections at 30, 60, 90 and 120 min post glucose ingestion.

#### Venous blood collection and analysis

For all time-points venous blood samples were collected in SST (5 mL; cholesterol, triglycerides and insulin) and fluoride oxalate (4 mL; glucose) tubes. All samples were centrifuged at 3500 rpm for 15 min at 4°C. Aliquots were frozen immediately at -20°C until analysis. At rest whole blood was refrigerated (4°C) in an EDTA tube (4 mL) for a maximum of 6 h until analysis of glycated haemoglobin (HbA1c).

Fasting venous blood samples were analyzed for total cholesterol (Enzymatic method and polychromatic endpoint technique), high-density lipoprotein (Accelerator selective detergent methodology), low-density lipoprotein (Friedwald Equation), triglycerides (Enzymatic method and biochromatic endpoint technique; Dimension Xpand Plus, Siemens Healthcare Diagnostics, Sydney, Australia) and HbA1c (%A1c) (High-performance liquid chromatography: Bio-Rad Variant, Bio-Rad Laboratories, Sydney, Australia). Samples collected from the OGTT were also analyzed for glucose (ABL825 Flex Analyzer, Radiometer Medical ApS, Bronshoj, Denmark) and insulin (Solid-phase chemiluminescent enzyme immunometric assay: Immulite 2000, Siemens Healthcare Diagnostics, Los Angeles, CA, USA) with an intra and inter-assay coefficients of variation between 4.0–7.4%. As an indication of Homeostasis Model Assessment: Insulin Resistance, (HOMA-IR) was calculated based on (fasting insulin x fasting glucose)/22.5 [26]. For the analysis of blood glucose metabolism, glucose and insulin Area Under the Curve (AUC) over the 2 h OGTT was calculated using previously validated methods [27] and estimated insulin sensitivity was calculated through the previously validated Matsuda-ISI model [28].

#### Muscle biopsy collection

For both pre and post-intervention muscle biopsies, it was ensured that participants were fasted state (10–12 h) and refrained from physical activity for 72 h prior to collection. Final training sessions were staggered across all groups to ensure accurate timing of the 72 h post-training muscle samples. After administration of a local anaesthetic (2% plain Lignocaine) a muscle biopsy was obtained from the lateral portion of the *m*. vastus lateralis of each participant. Using a 5-mm Bergstrom needle an ~100 mg sample was obtained, blotted on filter paper, removed of fat and connective tissue, frozen in liquid nitrogen and stored at -80°C until further analysis. Samples were analyzed for total protein content of PGC-1α (Calbiochem ST1202), nuclear respiratory factor (NRF)1 (Abcam ab34682), NRF2 (Santa Cruz sc-22810), mitochondrial transcription factor (Tfam; Abcam ab47517), mitochondrial complex I-V (Mitoprofile Human Total OXPHOS antibody cocktail, Mitosciences ab110411), myocyte enhancer factor 2A (MEF2A; Abcam ab87975), SIRT 1 (Cell Signaling 8469), GLUT4 (Millipore CBL242), p53 (Cell Signaling 2527), AKT (Cell Signaling 9272) and α-tubulin (Cell Signaling 2125), which was used as a loading control.

#### Western blotting analyses

Approximately 20 mg of frozen muscle was homogenized in 400 μL of ice-cold lysis buffer (50 mM Tris-HCI (pH 7.4), 1% Triton X-100, 0.1% SDS, 1μg/ml Aprotinin and Leupeptin, 1 mM Benzamidine, 1 mM NaF, 150 mM NaCI, 1mM EDTA, 5 mM Na-pyrophosphate, 1 mM DTT, 1 mM PMSF and 1 mM Na_3_ VO_4_). Samples were homogenized followed by end-over-end rotation for 60 min at 4°C. Homogenates were centrifuged at 15,000 g for 10 min at 4°C and supernatant was collected. The protein content of the supernatant was determined with a Bradford assay using a protein assay dye reagent and bovine serum albumin (BSA) as the standard. Each sample was diluted with equal volume 2X Laemmli buffer (125 mM Tris-HCI (pH 6.8; 4% SDS, 20% glycerol, 0.015% Bromophenol Blue) and β-mercaptoethanol (10%). For each blot an internal standard was loaded along with 10–25 μg of protein for each sample and separated in Tris-glycine running buffer using self-cast stacking 4% and 8–12% resolving gels. In transfer buffer, gels were transferred wet onto PVDF membranes for 90 min at 100 V. Membranes were blocked at room temperature (RT) by incubating for 1 h in 5% fat-free milk and Tris buffered saline 0.1% Tween-20 (TBST). Membranes were washed 3 x 5 min in TBST and then incubated with primary antibodies (dilutions based on the manufacturer’s instructions) in 3% fat-free milk or fatty acid-free BSA overnight (16 h) at 4°C.

**Fig 2:**
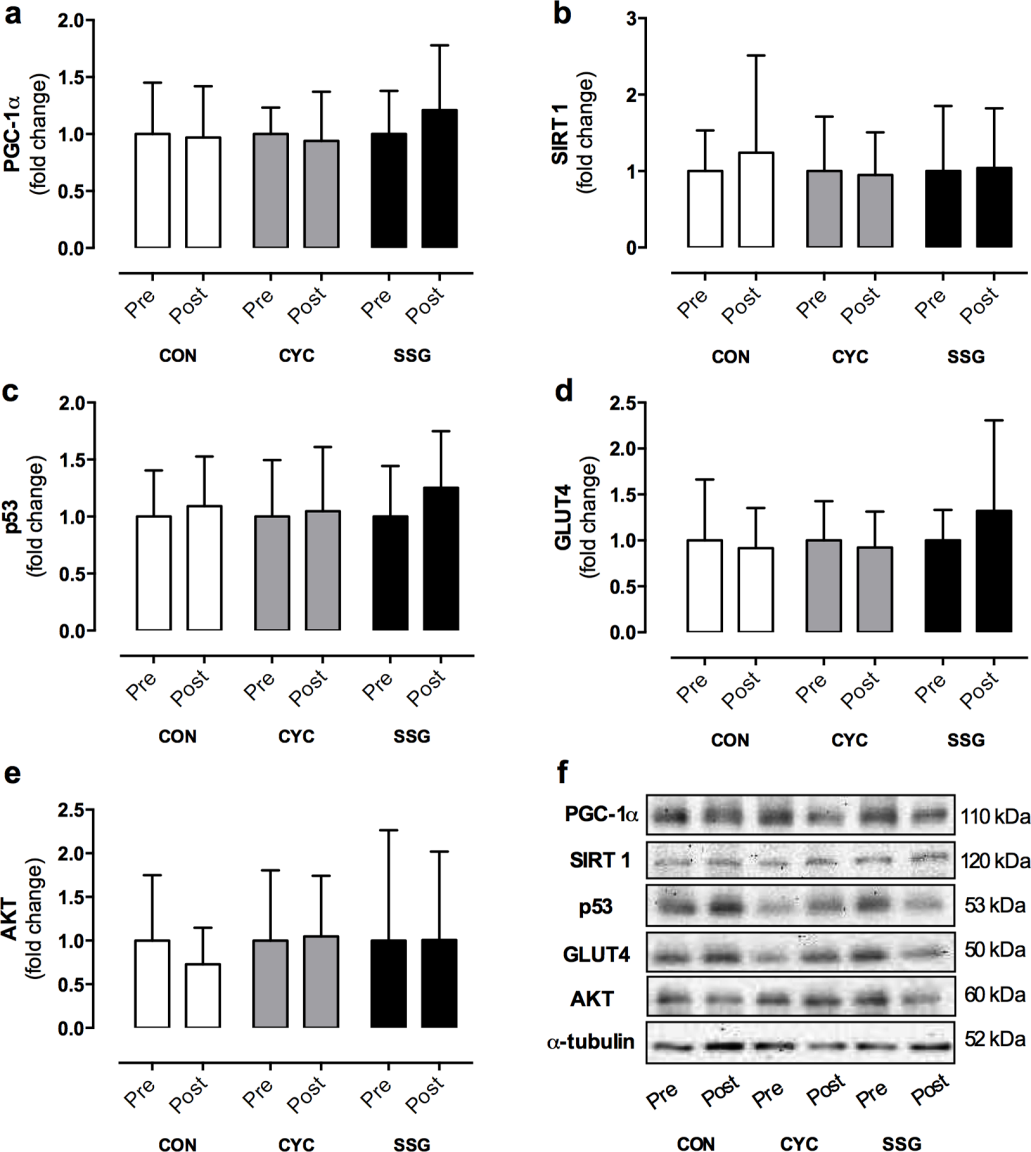
Total protein content of PGC-1α (a), SIRT-1 (b), P53 (c), GLUT4 (d), AKT (e) and representative blots corrected to α-tubulin (f) before (pre) and after (post) 8-weeks of control (CON; n = 9), continuous cycling (CYC; n = 11) or small-sided games (SSG; n = 10) conditions. Post values are expressed as fold change relative to pre values.

The following morning, membranes were washed for a further 3 x 5 min in TBST and incubated with anti-species horseradish peroxidise-conjugated secondary antibody (1:10000 dilutions) in 1% fat-free milk for 90 min at room temperature. After a further 3 x 5-min washes in TBST, membranes were exposed to a chemiluminescence liquid (2.5 mM Luminol, 400 μM p-coumaric acid, 100 μM Tris (pH 8.5), 5.4 mM H_2_O_2_) for 5 min. Membranes were visualized using a Versa Doc 4000 MP imaging system and band densities were determined using Quality One image-analysis software, Bio-Rad. Pre and post samples for each participant and a participant representative from each training group were run in the same gel. Raw densitometry data were used for statistical analysis, and for graphical purposes fold change relative to pre-training values are displayed in Figs 1 and 2.

### Statistical analyses

All data are reported as mean ± SD, and all statistical analyses were conducted on raw data points and the change in raw data values. Non-normally distributed variables (PGC-1α, SIRT1, p53, GLUT4, AKT, MEF2A, Tfam, NRF1, NRF2, mitochondrial complexes I-V, insulin AUC and glucose AUC) were log transformed prior to all analyses. A one-way repeated measure ANOVA (pre to post intervention) was used to compare baseline variables between conditions and the effects of each intervention for all measured variables with Tukey’s HSD post-hoc test. Two-way repeated measures ANOVA (pre to post intervention x 5 time points of glucose load) was used to assess the effect of each intervention on glucose and insulin. When a significant condition x time interaction occurred a post-hoc paired sample t-tests were used to determine where any difference lay pre- to post-intervention within each group. Significance was accepted at P<0.05. All statistical analyses were performed using PASW for MS-Windows version 20.0 (Statistical Package for the Social Sciences, Chicago, IL, USA). Insulin area under the curve was used for the power calculation. This variable has been reported to change 23.3% in response to 12 weeks of training [29]. This equated to a decrease of 2983 ±2536 μU^.^min^.^mL^-1^ for a 120-min OGTT. When calculating the power analysis these values were substituted with an alpha set at 0.05 and a power set at 80%. Eight participants per group were calculated as necessary to detect a significant change.

## Results

### Compliance and training load

Participant numbers for completion of the study (and as included in the pre and post training analyses) were CON (n = 11), CYC (n = 11) and SSG (n = 10; Fig 1). One participant could not complete the SSG training due to a knee injury sustained during training (week 3). Two participants in the control group did not consent to a post-training muscle biopsy, thus final numbers for muscle analysis were CON (n = 9), CYC (n = 11) and SSG (n = 10; Fig 1). There were no significant differences in attendance to training sessions between the groups with a mean of 91 ±2% for SSG and 95 ±2% for CYC (p = 0.127). Training intensity was comparable between conditions as represented by mean % HR_max_ (SSG, 85.3 ±1.1%; CYC, 84.5 ±1.3%; p = 0.641) and session-RPE (SSG, 11.3 ±0.4 AU; CYC 12.0 ±0.2 AU; p = 0.112; Table 2).

### Body composition

Results showed no significant changes within or between conditions for measurements of body mass, BMI, waist girth and WHR (p>0.05; Table 1). Changes within and between conditions for TB-FM, TB-FFM, and IA-FM are provided in Table 1. A significant condition-by-time interactions was evident for relative (p = 0.0001) and absolute TB-FM (p = 0.002). There was a significantly greater effect for CYC (relative (%) p = 0.0001; Absolute (kg) p = 0.004) and SSG (relative (%) p = 0.001; absolute (kg) p = 0.005) compared to CON, and SSG and CON. A significant condition-by-time interaction was evident for TB-FFM (p = 0.017), with a greater effect for CYC (p = 0.04) and SSG (p = 0.013) compared to CON. A significant condition-by-time interaction was evident for IA-FM (p = 0.001), with a significant greater effect for CYC (p = 0.001) and SSG compared to CON (p = 0.015).

### Oral glucose tolerance test and fasting blood chemistry

Results from the OGTT, estimated insulin sensitivity, and fasting blood glucose, insulin and cholesterol are provided in Table 3. There were no significant changes within or between conditions for fasting blood glucose, insulin and cholesterol values (p>0.05). A significant condition-by-time interaction was evident for HbA1c (%A1c; p = 0.034), with a significantly greater effect for SSG compared to CON (p = 0.009). A significant condition-by-time interaction was evident for estimated insulin sensitivity (Matsuda ISI; p = 0.002), with a significant greater effect for CYC (p = 0.009) and SSG compared to CON (p = 0.002). A significant condition-by-time interaction was evident for glucose AUC (p = 0.008), with a significant greater effect for SSG (p = 0.021) and CYC (p = 0.004) compared to CON. A significant condition-by-time interaction was evident for insulin AUC (p = 0.002), with a significant greater effect for SSG (p = 0.001) and CYC (p = 0.024) compared to CON.

**Table 3:** Mean ± SD fasting blood chemistry and glucose tolerance test pre and post 8 weeks of control (n = 11), continuous cycling (n = 11) or small-sided games (n = 10).

	Control		Cycling		Small-Sided Games	
Variable	Pre	Post	Pre	Post	Pre	Post
Glucose (mmol L^1^)	4.8 ± 0.9	4.8 ± 0.8	4.8 ± 0.7	4.7 ± 0.7	4.7 ± 0.6	4.4 ± 0.6
Insulin (μlU mL^1^)	8.8 ± 6.6	10.1 ± 8.3	8.5 ± 2.4	7.8 ± 7.2	7.5 ± 6.9	5.3 ± 3.8
Glucose AUC (mmol^.^L^1^ (120 min)^1^)	12.3 ± 1.5	13.1 ± 2.7	14.1 ± 3.7	11.9 ± 3.9 ^a^ ^,^ ^b^	12.7 ± 3.1	10.4 ± 1.5 ^a^ ^,^ ^b^
Insulin AUC (μlU^.^mL^1^ (120 min)^1^)	111.6 ± 74.1	140.7 ± 85.2 ^a^	108.5 ± 78.4	104.6 ± 75.1	102.1 ± 75.0	71.8 ± 40.7 ^a^ ^,^ ^b^
Matsuda ISI (μlU mL^1^, mg mL^1^)	9.4 ± 7.3	6.7 ± 4.2	8.5 ± 6.7	11.3 ± 9.6 ^a^ ^,^ ^b^	8.7 ± 5.8	11.8 ± 5.7 ^a^ ^,^ ^b^
HOMA-IR (μlU mL^1^, mmol L^1^)	2.0 ± 1.7	2.3 ± 2.2	1.9 ± 2.0	1.8 ± 1.8	1.7 ± 1.7	1.1 ± 0.8
HbA1c (%A1c)	5.5 ± 0.4	5.5 ± 0.4	5.7 ± 0.7	5.4 ± 0.4 ^a^ ^,^ ^b^	5.7 ± 0.4	5.4 ± 0.3 ^a^ ^,^ ^b^
Total Cholesterol (mmol L^1^)	5.1 ± 1.0	5.1 ± 0.7	5.3 ± 0.8	5.1 ± 0.8	5.4 ± 1.0	5.2 ± 0.9
HDL (mmol L^1^)	1.3 ± 0.4	1.2 ± 0.4	1.3 ± 0.2	1.3 ± 0.3	1.2 ± 0.2	1.2 ± 0.3
Triglycerides (mmol L^1^)	1.1 ± 0.7	1.3 ± 0.5	1.3 ± 0.6	1.3 ± 0.6	1.4 ± 0.7	1.5 ± 0.7
Hazard Ratio (Total: HDL)	3.9 ± 1.0	4.2 ± 1.1	4.3 ± 1.1	4.2 ± 1.3	4.7 ± 1.4	4.5 ± 1.5

AUC, Area under the curve; Matsuda ISI, Estimate of insulin sensitivity; HOMA-IR, Glucose homeostasis—insulin resistance; HDL, High density lipoproteins.

^a^ = Significant within group change (p<0.05)

^b^ = Significant change compared to control group (p<0.05)

### Oxygen consumption and strength

Changes to upper- and lower-body strength, and aerobic capacity variables (VO_2_, test duration and power output at 80% HR_max_), are provided in Table 1. A significant condition-by-time interaction was evident for VO_2_ at 80% HR_max_ (p = 0.0001) with a significant greater for CYC (p = 0.0001) and SSG (p = 0.0001) compared to CON. This corresponded to a significant condition-by-time interaction for test duration (p = 0.0001), with a greater effect for CYC (p = 0.0001) and SSG compared to CON (p = 0.002). Power output (Watts) showed a significant condition-by-time interaction (p = 0.001) with a greater effect for CYC (p = 0.0001) and SSG (p = 0.007) compared to CON. Chest press showed no significant changes within or between all conditions (p = 0.388). Leg-press showed a significant condition-by-time interaction (p = 0.005), with a greater effect for SSG compared to CYC (p = 0.045) and CON (p = 0.008).

### Skeletal muscle protein content

**Fig 3:**
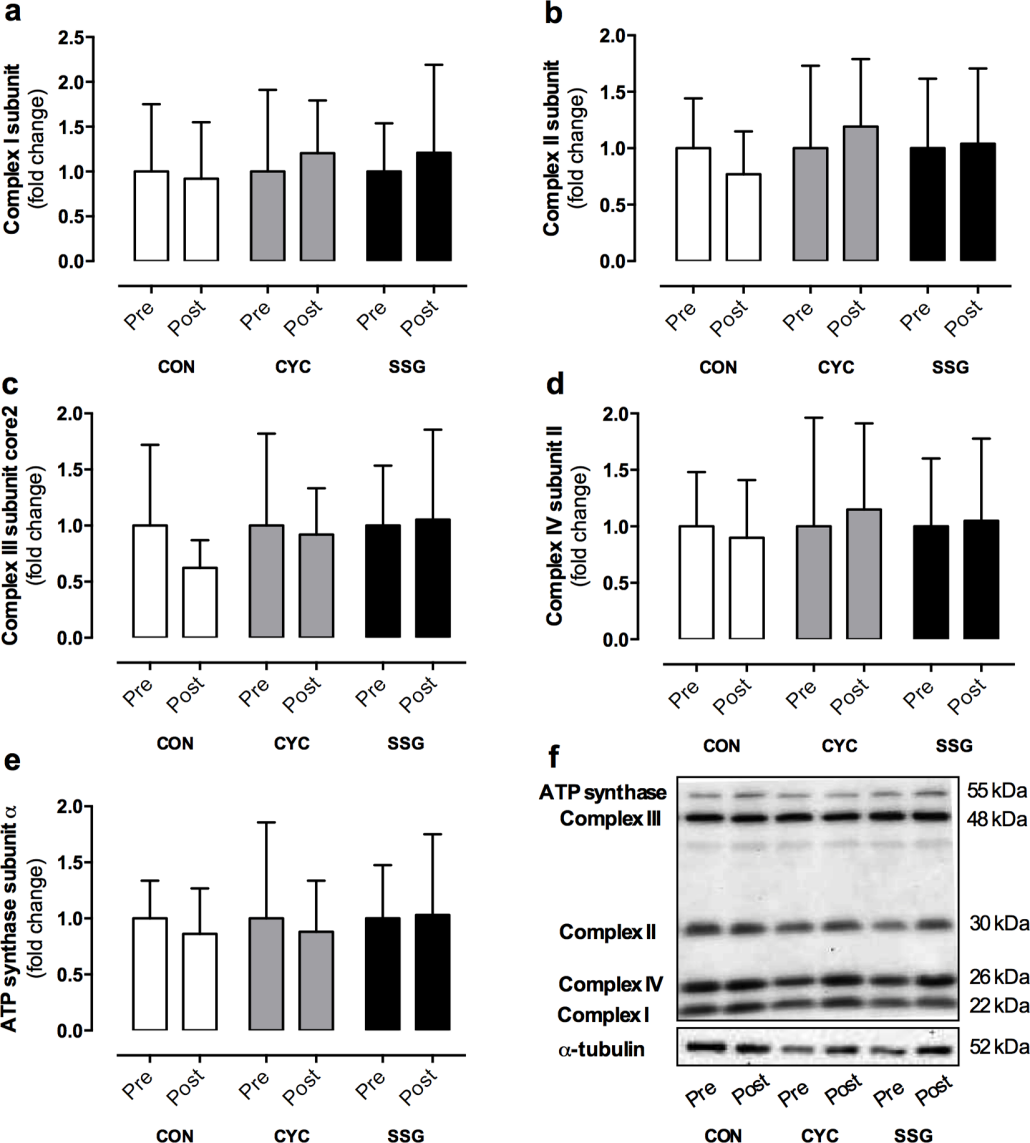
Total protein content of Mitochondrial Complex I subunit (a), Complex II subunit (b), Complex III subunit core2 (c), Complex IV subunit II (d), ATP synthase subunit-α (e) and representative blots corrected to α-tubulin (f) before (pre) and after (post) 8-weeks of control (CON; n = 9), continuous cycling (CYC; n = 11) or small-sided games (SSG; n = 10) conditions. Post values are expressed as fold change relative to pre values.

Results for PGC-1α, SIRT-1, P53, GLUT4 and AKT are shown in Fig 2. Mitochondrial complexes I-V are reported in Fig 3. All data are reported as fold change relative to the pre-training values. There were no significant changes within or between conditions in any of the respective proteins as a result of the 8-week intervention (p>0.05). The transcription factors MEF2A (CON, 0.69 ±0.71; CYC, 0.90 ±0.69; SSG, 1.06 ±0.96 AU; p>0.05), Tfam (CON, 0.82 ±0.51; CYC, 0.81 ±0.75; SSG, 0.74 ±0.51 AU; p>0.05), NRF1 (CON, 0.98 ±0.69, CYC, 1.12 ±0.63; SSG, 0.69 ±0.37 AU; p>0.05) and NRF2 (CON, 0.84 ±0.47; CYC, 1.26 ±0.76; SSG, 0.78 ±0.68 AU; p>0.05) also showed no significant changes in response to the exercise training program (p>0.05). α-tubulin was used as a loading control and showed no significant changes within or between conditions following training (p>0.05).

## Discussion

The present study examined the efficacy of rugby SSG as an initiative to improve risk-factors associated with the prevention of T2DM. The main findings revealed that 8 weeks of either SSG or CYC training induced comparable improvements in TB-FM, aerobic capacity, estimated insulin sensitivity and glucose AUC. Although training was matched for internal load and duration, SSG was the only condition to decrease insulin AUC, and to increase TB-FFM and leg strength. Furthermore, the control group showed an increase in TB-FM, IA-FM and insulin AUC. Despite these improvements in clinical risk-factors for the SSG and CYC conditions, skeletal muscle proteins associated with mitochondrial biogenesis and glucose metabolism showed no significant changes.

Common measures utilized by health professionals for the diagnosis of metabolic disease include HbA1c (%A1c), fasting glucose and insulin, estimated insulin resistance (HOMA-IR), estimated insulin sensitivity (Matsuda ISI) and/or an OGTT [2, 28]. Consistent with the current findings, 12 weeks of SSG (soccer) training has been reported to have no significant effect on fasting glucose concentration [9, 30]. In the present study, there were also no significant changes in HOMA-IR, which was reflected by non-significant changes in fasting glucose and insulin concentrations within all conditions. However, as all participants were normo-glycemic during the pre-intervention period, the likelihood for exercise training to improve resting glucose and insulin concentrations was reduced [2]. In contrast, both training interventions resulted in a decrease in HbA1c. This magnitude of decline in HbA1c in both groups was similar to that previously reported following >12 weeks of moderate-intensity endurance exercise training [31]. The present study is the first to show that SSG training can reduce HbA1c concentration, with the decrease similar to that occurring following traditional, continuous aerobic training. The decrease in HbA1c in both training groups should decrease the development and progression of microvascular complications [32]. For example, a 1% A1c rise above 5% A1c increases the risk of all-cause mortality and cardiovascular death by 41% [32]. These relationships indicate that both SSG and CYC conditions improved long-term glycemic control and decreased the risk of a future cardiovascular event [32].

The decreased HbA1c and training-induced increase in insulin sensitivity (Matsuda ISI) in both groups of the present study signifies improved glucose metabolism [33]. These results also coincided with a similar reduction for glucose AUC in both training groups. Of particular interest, SSG was the only condition to show a decrease in insulin AUC. Thus, for the first time we report that SSG is an effective training mode that can be used for improving glucose disposal and reducing insulin release in response to a standard glucose load. The results of SSG training are consistent with literature involving SIT and futsal, which has been shown to lower glucose and insulin concentrations during an OGTT [10, 34]. Although the results between SSG and previous SIT training studies are similar, the social and community-based benefits of SSG may lead to better adherence and compliance within an inactive middle-aged cohort [5].

The preservation of strength and muscle mass through exercise training can also help ameliorate metabolic dysfunction and prevent the onset of T2DM [5, 35]. In the present study, SSG training led to an increase in TB-FFM and leg strength compared to no change in either the CYC or CON groups. Previous interventions involving SSG training (12 weeks) have reported increases in maximal isometric hamstring strength and lean muscle mass, when compared to continuous running, within an inactive cohort [5, 12, 36]. These previous findings, along with those of the present study, suggest that SSG training provides sufficient loading to induce myofibrillar protein synthesis, skeletal muscle hypertrophy and an increase in leg strength. These aforementioned adaptations in the SSG condition may also provide a mechanism for the observed decrease in insulin AUC that was not observed in CYC [37], although further research is required to assess these specific hypotheses. Importantly, the increased leg strength and TB-FFM indicates a potential advantage of SSG training over continuous, aerobic training to improve glucose metabolism and reduce risk-factors associated with T2DM [35, 37].

Abdominal adiposity is also correlated with the development of cardiovascular and metabolic diseases, with this relationship persisting after accounting for the effects of increased TB-FM [38]. In the present study, TB-FM deceased by 3.6% in SSG and 2.9% in CYC. These reductions are similar to previous observations following 12 weeks of SSG (soccer) training in inactive men, which report a decrease in fat-mass by 3.0%, compared to a 1.8% decrease with continuous running [13]. In the present study, there was also an interaction effect of IA-FM for both training conditions, compared to CON; within all groups no significant changes were evident in body mass, BMI or WHR. Regardless of the small changes in anthropometry, SSG training was equally effective as CYC at improving TB-FM and IA-FM, which are two important risk-factors associated with the development of T2DM [38].

Previous research has demonstrated a greater increase in VO_2max_ with SSG (soccer, 13%) compared to continuous running (8%) [8, 13]. In comparison, the current study observed similar increases in sub-maximal VO_2_ between conditions (19.1% in CYC and 18.9% in rugby SSG). This increase in VO_2_ coincided with an increase in test duration and workload at 80% HR_max_ (Table 1). These results indicate that SSG is as effective as CYC for inducing adaptations in aerobic capacity within a previously inactive population, especially when training is matched for internal training load. From a clinical perspective, men with high cardiorespiratory fitness (>35.7 mL^.^kg^-1.^min^-1^) have been shown to be nearly two-thirds less likely to develop metabolic syndrome [3]. These improvements in aerobic capacity have the potential for preventing the development of metabolic syndrome and associated co-morbidities [3].

Despite the positive adaptations of the aforementioned clinical risk factors within the present study, there were no significant changes in the content of skeletal muscle proteins associated with glucose regulation and mitochondrial biogenesis (Figs 2 and 3). Improvements in insulin sensitivity and glucose uptake have been shown to be facilitated by increased GLUT4 and AKT content [9, 14, 15]. An important function of AKT is to mediate the metabolic actions of insulin to stimulate cellular glucose transport [39, 40]. Given that GLUT4 is highly abundant in skeletal muscle and is associated with enhanced glucose disposal and insulin action, there has been extensive interest in therapeutic strategies to increase AKT and GLUT4 expression in cohorts at risk of developing metabolic disorders [39, 40]. To our knowledge, there are no other published findings that have investigated changes in the content of proteins associated with glucose regulation in response to rugby-specific SSG training. In the present study, both SSG and CYC training were associated with favourable changes in blood chemistry relating to glucose disposal (OGTT); however, there were no corresponding increases in GLUT4 or AKT protein content. One possible explanation is that the improved insulin sensitivity with exercise may be more dependent on increased translocation of GLUT4 to the cell surface, rather than an increase in GLUT4 abundance [41]. As we did not measure GLUT4 translocation in the present study, further research is required to test this hypothesis.

This is the first study to investigate adaptations of mitochondrial complexes I-V in response to SSG, with both training groups showing no significant effect on the protein abundance of these complexes (Fig 3). Both SIT and aerobic training have been associated with increases in the expression and activity of COX II and IV in healthy trained adults [42, 43]; however, an increase [44] or no change [45] has also been reported in inactive adults. As the intensity and duration of exercise has a direct effect on COX activity and expression [46], it appears that the accumulated training stimulus of SSG and CYC was below the threshold required to significantly increase the protein content of these mitochondrial complexes. An additional observation from the present study is that adaptations in pulmonary measures of VO_2_ were not reciprocated by any observed changes in total protein content of mitochondrial complex (I-V) within skeletal muscle. Improvements in aerobic capacity are also related to improved cardiovascular function, vascular content and resistance [36]. Accordingly, the absence of significant changes in the protein content of the mitochondrial complexes (I-V) in the present study suggest that the predominant training adaptations were cardiovascular [6], and/or adaptations of mitochondrial functioning/efficiency [4, 43]. However, further research is required to verify these hypotheses.

Given the absence of significant changes in the protein content of the mitochondrial complexes, it is not surprising that there were no significant changes in other proteins associated with mitochondrial biogenesis. Both SIRT1 and p53 are two of the many proteins that have been reported to acutely regulate PGC1-α, and thus may contribute to exercise-induced mitochondrial biogenesis [16, 17, 43]. In the present study, there were no significant changes in SIRT1, p53 or PGC1-α protein content; in addition there were no changes in associated transcription factors (Tfam, NRF1, NRF2 and MEF2A) after either training intervention. There is limited literature available on the SIRT1 response to exercise in humans, with no reports of changes in the content of proteins associated with mitochondrial biogenesis following SSG training. A 16% increase in PGC-1α protein content has been reported following 6 weeks of interval training [17]. Interestingly, in the same study there was a 20% decrease in SIRT1 protein content, but a 31% increase in SIRT1 activity [17]. In contrast, an increase in SIRT1 (56%) content, no changes in PGC-1α content, and an increase in PGC-1α nuclear abundance (~25%) has been reported in response to SIT training [43]. More recently, using rat models, p53 has emerged as a potential acute regulator of mitochondrial content and function [16], although there are no published studies involving exercise training in humans. As such, the measurement of p53 provides an additional novel element to the current study even though there was no change of total protein content in response to either SSG or CYC training within an inactive population. Similar to the results of the present study, no significant changes in mitochondrial protein content in response to exercise training (aerobic and/or resistance, intermittent) has previously been reported in sedentary and active cohorts [45, 47, 48]. In addition to these previous reports [45, 47, 48], results of the current study suggest that longer (>12 weeks) training programs may be required in previously inactive men to promote increases in skeletal muscle protein content associated with mitochondrial biogenesis and glucose regulation.

### Limitations

Despite the potential glucose regulatory benefit of exercise, some limitations in the present study should be acknowledged. Firstly, although the number of participants was typical for muscle biopsy studies, it could be conceived as relatively low, and the effect of this on the statistical power of the muscle analyses is accepted as a limitation. Further, a sub-maximal VO_2_ test was conducted and thus the ability to compare results to previous studies measuring VO_2max_ is compromised. However, this is unlikely to affect the findings of the current study as sub-maximal tests are well recognised as appropriate and sensitive measures of aerobic fitness. Additionally, the use of age-predicted (220 bpm—age) HR_max_ is not anchored against a true individual HR_max_, which may create differences in relative intensity between participants and the determination of sub-maximal VO_2_ during the GXT, and hence should be acknowledged as such. However, it should be noted that the use of age-predicted HR_max_ and sub-maximal VO_2_ are sensitive to experimental manipulation and therefore appropriate for assessing training adaptation in aerobic function. Although group allocation was randomized, complete elimination of all bias may not be possible, and the data should be interpreted accordingly. Moreover, caution is advised in the interpretation of fasting blood chemistry and OGTT-derived results, as it is acknowledged the low reproducibility of this technique can result in discrepancies in findings. Finally, the knee injury sustained as results from SSG training may be considered as a limitation with this training method and further data on longitudinal effects of such training on orthopaedic injuries should be considered.

In conclusion, for the first time, our results indicate that SSG training is an effective alternative to continuous cycling for improving metabolic risk-factors associated with the prevention of T2DM. The current study revealed improvements in glycemic control, glucose AUC, aerobic capacity, abdominal and total-body fat-mass in response to both CYC and SSG training, while SSG showed additional improvements in insulin AUC, lean muscle mass and lower-body strength. Despite these improvements in response to CYC and SSG training, there were no changes in the content of skeletal muscle proteins associated with glucose regulation and mitochondrial biogenesis. As such, additional research should focus on longer (>8 weeks) training programs to investigate adaptations in skeletal muscle protein content in middle-aged, inactive men. Given previous reports of greater motivation and enjoyment associated with participation in team sports, the incorporation of SSG into an exercise training approach may encourage long-term compliance for increased levels of physical activity and the overall prevention of T2DM [5].

## Supporting Information

S1 File CONSORT 2010 Checklist. CONSORT 2010 checklist for reporting information of a randomized trial.
(DOCX)

S2 File Protocol as submitted to ethics. Research methodology and procedures as submitted to ethics.
(DOCX)

S3 File Approved IRB documents. Informed consent form and information sheets as submitted to ethics
(DOCX)

S4 File Raw data. Tables reporting raw data for all primary and secondary variables assessed.
(PDF)
