# Supplementary material for: Rugby-Specific Small-Sided Games Training Is an Effective Alternative to Stationary Cycling at Reducing Clinical Risk Factors Associated with the Development of Type 2 Diabetes: A Randomized, Controlled Trial
Source: PLoS One. 2015 Jun 1;10(6):e0127548. doi: 10.1371/journal.pone.0127548 (PMC4452519; doi:10.1371/journal.pone.0127548)
Supplement: S2 File — Research methodology and procedures as submitted to ethics. (DOCX) [file pone.0127548.s002.docx]

**Title of Research Project**

| “Effects of 8 weeks aerobic or modified football training on skeletal muscle markers of mitochondrial functioning, systemic inflammation and glucose regulation.” |
| --- |

**Methodology/Procedural Evaluation**

| - 1. **Describe the research procedures/methodology *as they affect the research participants and any other parties involved*.**   **Include:**   - Step by step account of each stage in the research plan that will affect participants (and any relevant others)   - Note that these stages begin with recruitment and may extend as far as data analysis - Justification of the research approach |
| --- |
|  |

*Subject Population*

The study population will comprise of 36 sedentary, Caucasian male subjects aged 35-55 y, who are in a non-diseased state. All testing procedures and training programs will be conducted at the CSU laboratory and subjects will be recruited from regional Bathurst areas. All subjects will be randomized into exercise protocols including an aerobic, modified football, or control protocol, allowing 12-15 subjects in each protocol to complete the allocated training intervention. Subject recruitment will ensure a non-smoking sample population group representative of a sedentary lifestyle, but not clinically diagnosed with any pre-existing CVD or metabolic disorders. Participants with orthopaedic limitations will also be advised against study involvement due to the musculoskeletal demands of the respective exercise protocols. All participants will provide verbal and written consent prior to engagement in testing procedures and Human Ethics clearance will be granted by the CSU EHRC and RSC.

**Pre- and Post-Intervention Testing**

Participants will be required to attend two testing session prior to the commencement of exercise training, and two testing sessions after the 8 week intervention period. An outline of the testing sessions and training program is provided in the below sections.

*Anthropometry and DXA*

During pre- and post-intervention testing, mass, height, waist and hip girth measures will be measured to calculate BMI (mass [kg]/ height [m^2^]), and waist to hip ratio (WHR). Additionally, subjects will undertake supine whole bodies DXA scan (XR800, Norland, Cooper Surgical Company, USA) for the determination of total and regional body composition, for analysis of abdominal adiposity, total body fat mass and total body lean mass. Ionising radiation will be used as part of the DXA scan. Each scan will be conducted at a scanning speed of 130mm/sec and the scanning resolution will be 6.5 x 13.0mm, and the dual energy x-ray beam is in the range 35-90KeV.

Self-Reported Questionnaire

All participants will be administered the *MOS SF-36 questionnaire.* The MOS SF-36 (McHorney et al., 1993) contains eight sections assessing self-reported physical function, problems experienced with work or daily activities based on physical health, bodily pain, problems experienced with work or daily physical activity based on emotional health, social functioning, general health, vitality and mental health (see Appendix). This questionnaire will provide a holistic approach to the benefits of an exercise training program within a rural Aboriginal population, potentially elucidating regular exercise with beneficial changes in physical, functional and mental health.

*Sub-Maximal Aerobic Capacity Testing*

Subjects will perform a sub-maximal graded exercise test (GXT) on an electronically braked cycle ergometer (LODE Excalibur Sport, LODE BV, Groningen, The Netherlands) to determine oxygen consumption and maximal aerobic power output. The test will incorporate an incremental step protocol consisting of 1 min stages for each step of the protocol, commencing at 25 W with increments of 25 W each minute. Heart rate (Vantage NV, Polar, Finland) will be recorded each minute throughout the protocol, and subjects exercised until volitional exhaustion or upon attainment of 80% age-predicted maximal heart rate (MHR). This aerobic fitness test will familiarise participants to the aerobic cycling protocols and will identify an initial starting point for week one of training.

*Sub-Maximal Strength Testing*

Following a 30 min recovery period from maximal aerobic testing subjects will complete a 3RM strength test. The 3RM testing procedure will identify upper-body and lower-body strength of seated chest press and leg press (Panatta Sport, Apiro, Italy), respectively. Subjects will attempt ascending resistances, separated by a 2 min recovery period until the determination of upper- and lower-body 3RM. Individual seating positions of head rest, back rest and seat height will be recorded and replicated during post-intervention testing.

*Oral Glucose Tolerance Test (OGTT)*

Following sufficient rest, a 2h OGTT will be administered (according to standardised research procedures; Katzel et al., 1997; Baldi et al., 2003). Participants will have a 20GA in-dwelling catheter inserted by a trained phlebotomist into an antecubital vein for periodic blood sampling during the OGTT. A standardised 75 g oral glucose solution will be ingested (within 5 minutes) following a 10-12 h overnight fast. Following ingestion, venous samples will be collected at the 30, 60, 90 and 120 min time-points for the analysis of insulin, glucose and C-peptide.

*Venous Blood Collection*

Subjects will refrain from any physical activity or exercise for 48 h prior to testing and the consumption of alcohol and caffeine 24 h prior to each data collection session. During testing subjects will arrive in a fasted state for the collection of venous blood for analysis of CRP, fasting blood glucose, fasting insulin, HbA1c and total lipid profile (total cholesterol, LDL, HDL, Triglycerides), TNF-α, IL-6, Il-1β and IL-1ra. Additionally, insulin, glucose and C-peptide will be measured during the OGTT. All venous blood samples will be collected from a medial antecubital vein and centrifuged at 3500 rpm at 4^◦^C for 15 min. Samples will be sent for immediate analysis or aliquots will be immediately frozen and stored at -80°C for later analysis.

*Muscle Biopsy Collection and Analysis*

Upon arrival participants will be greeted and escorted to a specially prepared bed station where they will be introduced to the Medical Physician who will be performing the biopsy procedure (as per previous CSU EHRC approval ref. no. 2010-23). The Physician will verbally overview the procedure with each participant and will confirm with each participant as to whether they have an allergy with anaesthetics. If the participant has no allergies the procedure will begin, where a biopsy site will be measured at a point ~15cm distal (superior) to the patella on the anterior surface of the thigh (superficial to the vastus lateralis muscle). The site will be shaved with a sterile razor (new razor for each participant) and an antiseptic foam. Following sterilization of the site, a local anaesthetic (Xylocaine 1%) will be administered with a 23GA needle and 5ml syringe. Following sufficient time as to ensure numbness of the site (~10 minutes), an incision will be made with a sterile size 11 scalpel, and a sterilized (solvent cleaned + autoclave) 6mm Bergstrom Biopsy needle will be used to excise a ~80mg piece of muscle (about the size of a split pea). Post-collection, the site will be cleaned and sterilized, steri-strips applied, and padded bandages and wound dressings will be fitted. Each participant will then rest for ~15-20 minutes while an ice-pack is applied over the site. Participants will have one resting biopsy taken pre and post-intervention.

**Exercise Training Programs and Control Condition**

Physical Activity and Nutritional Documentation

All participants will be provided with an exercise diary for the documentation of physical activity external to the study. Additionally, an exercise log will be kept by the Chief Investigator, which will consist of exercise information pertaining to the actual work completed during training sessions. Participants will also be informed of the importance of maintaining normal diet and nutritional patterns including macro- and micro-nutrient consumption (contribution from CHO, fat, protein; vitamin/mineral supplementation), time of consumption (lateness of evening meal), quantity of serving (dietary caloric restriction), type of preparation (cooking oils used, boil/steam/raw, etc), and alcohol consumption (type and quantity).

***The cycling and modified football training programs will consist of 4 mesocycles (blocks) of 2 weeks, with session duration and intensity steadily progressing from one mesocycle to the next. This protocol will allow for appropriate training adaptions to occur prior to an increase in intensity, thus allowing all participants to complete all exercise sessions in a comfortable manner.***

Aerobic (cycling) Training Program

The aerobic exercise program will involve participants performing continuous a cycling exercise on stationary ergometers for a total of 3 sessions per week. During the 8 weeks, exercise duration will increase from 30 to 45 min, and intensity will be increase from 70 to 80% of maximal heart rate. In each training session pedalling resistance, rpm and heart rate will be recorded at 5 minute intervals and at the conclusion an overall RPE will be obtained. See table below for progression, duration and intensity of the respective aerobic exercise program.

Modified Football Training Program

The modified football program will involve participants exercising for 3 sessions per week of touch football games. Additionally, during one training session per week GPS units will be utilised to identify any improvements in velocity and total distance covered throughout the exercise program. During the 8 weeks, total session duration will increase from 40 to 50 min, and total game play (intensity) will increase from 22 to 38 minutes. In each training session heart rate will be recorded during the rest intervals and at the conclusion an overall RPE will be obtained. See table below for progression, duration and intensity of the respective modified football program.

| Aerobic Exercise Program | | | | |
| --- | --- | --- | --- | --- |
|  | Wk 1 – 2 | Wk 3 – 4 | Wk 5 – 6 | Wk 7 – 8 |
| Intensity (%MHR) | 80-85 | 80-85 | 80-85 | 80-85 |
| Session duration* | 40 | 45 | 45 | 50 |
|  | | | | |
| Modified Football Program | | | | |
|  | Wk 1 – 2 | Wk 3 – 4 | Wk 5 – 6 | Wk 7 – 8 |
| Session Duration* | 40 | 45 | 45 | 50 |
| No. Rest Periods | 3 | 3 | 3 | 3 |
| No. Players | 6v6 | 6v6 | 6v6 | 6v6 |
| Field Size | 40m; 60m | 40m; 60m | 40m; 60m | 40m; 60m |

MHR = maximum heart rate; * = duration in minutes; No. = number;

Field Size = width (meters); length (meters); 6v6 = six-a-side, total 12

Players on field

Control Condition

The control condition will involve participants continuing their sedentary life and normal dietary and nutritional patterns for the 8 week intervention period. Participants will be provided with a dietary journal and a physical activity journal in which they will be required to document any dietary changes or physical activity which will include walking, strenuous manual labour, strenuous gardening, etc., respectively. Participants will receive both verbal and written instruction expressing the importance of maintaining these patterns, and the journals will be reviewed by the study investigators to ensure conformity with the control condition. Following the 8 week intervention period, participants within the control group will be invited to attend the CSU gymnasium to commence a specifically tailored exercise training program.
