# Supplementary material for: Rugby-Specific Small-Sided Games Training Is an Effective Alternative to Stationary Cycling at Reducing Clinical Risk Factors Associated with the Development of Type 2 Diabetes: A Randomized, Controlled Trial
Source: PLoS One. 2015 Jun 1;10(6):e0127548. doi: 10.1371/journal.pone.0127548 (PMC4452519; doi:10.1371/journal.pone.0127548)
Supplement: S3 File — Informed consent form and information sheets as submitted to ethics (DOCX) [file pone.0127548.s003.docx]

amendham@csu.edu.au

Mob: 0402 456 041

Fax: +61 2 6338 4579

www.csu.edu.au/faculty/educat/human

FACULTY OF EDUCATION

School of Human Movement Studies


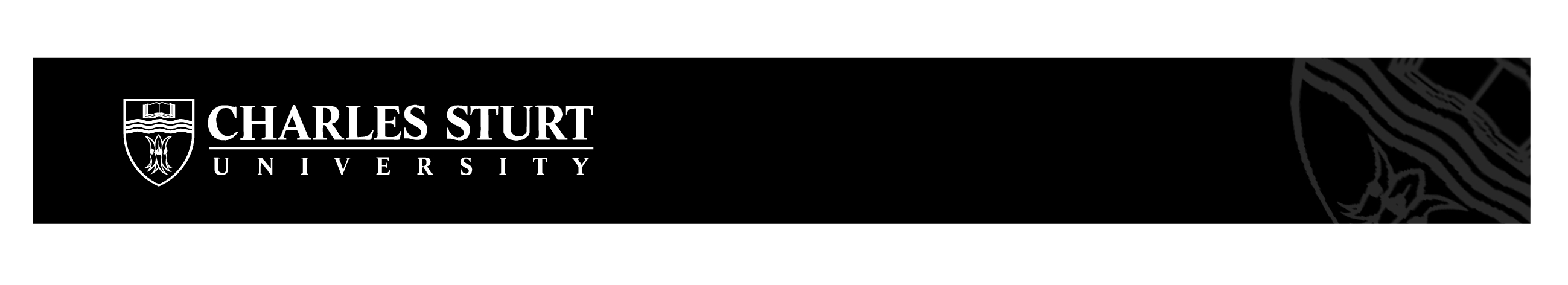


**Informed Consent Form**

**The effects of 8 weeks aerobic or modified football training on skeletal muscle markers of mitochondrial functioning, systemic inflammation and glucose regulation.**

Investigator Responsibilities - Participants Rights

1) As a subject you are free to withdraw your consent to participate at any time.

2) The researchers will answer any questions you may have in regard to the study at any time.

Questions concerning the study can be directed to:

Miss Amy Mendham Dr Rob Duffield Dr Aaron Coutts

PhD Student

Chief-Investigator Principal Supervisor Supervisor

Ph 0402456041 ph 63384939 Ph 0427 652 815

School of Human School of Human Leisure, Sport & Tourism

Movement Studies Movement Studies University of Technology

Charles Sturt University Charles Sturt University Sydney

***This study is part of PhD research by Amy Mendham, who is a PhD student at CSU.***

I have been provided verbally (by the Chief Investigator and/or Supervisor/s) and in writing (through the Study Information Sheet) with sufficient information on the study, including:

- My right to participate in this project, realising that I can withdraw at any time without being subject to any penalty or discriminatory treatment.
- My actual involvement and requirements in both the testing procedures and exercise training procedures within this study.
- Any potential risks or discomforts that I may experience during both testing procedures and exercise training procedures.
- How my confidentiality will be preserved, and how data collected from me will be used during and after my involvement in this study.
- Following express permission, your general practitioner may be contacted to ensure you are free from known disease*.*
- The use of any video footage or photographs of me taken during testing procedures or exercise training procedures.

🞎 I do not want video footage or photographs of me collected, stored, published, or used in any way.

I, (print your name) ________________________________ have read the information contained within this consent form and any questions I have asked have been answered to my satisfaction.

**_____________________ ______________**

**Signature of Participant Date**

**_____________________ ______________**

**Signature of Chief Investigator Date**

If any participants have any complaints regarding the manner, in which a research project is conducted, it may be given to the researcher or, alternatively to the Executive Officer, Ethics in Human Research Committee, Charles Sturt University, Bathurst, NSW (ph 6338 4628). All study participants will be provided with a copy of the Information Sheet and Consent Form for their personal records. This study has been approved by the Ethics in Human Research Committee, CSU, Bathurst, NSW.

amendham@csu.edu.au

Mob: 0402 456 041

Fax: +61 2 6338 4579

www.csu.edu.au/faculty/educat/human

Panorama Avenue

Bathurst NSW 2795

Australia

FACULTY OF EDUCATION

School of Human Movement Studies


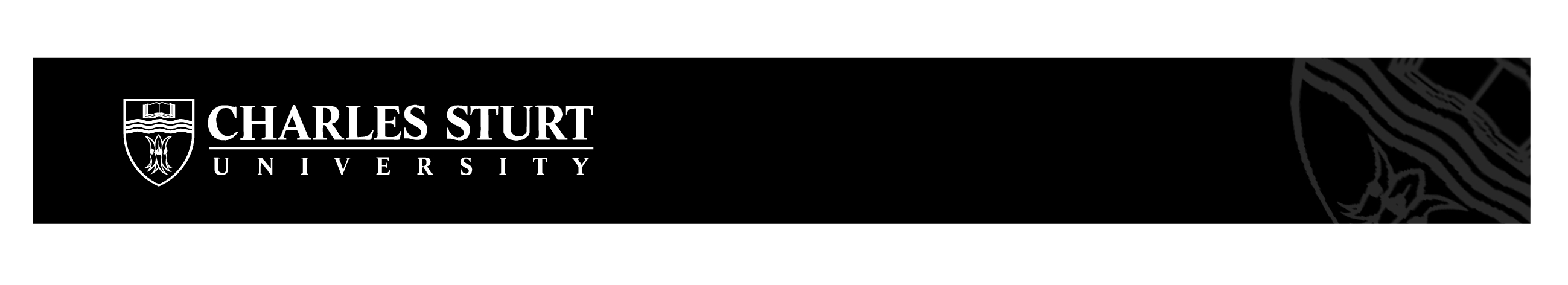


**INFORMATION SHEET**

**This study is part of PhD research by Amy Mendham, who is a PhD student at CSU.**

**Contact Details:**

If participants have any queries before, or throughout the testing and training procedures, please contact the Chief Investigator or Supervisor on:

Miss Amy Mendham

PhD student

Ph 0402 456 041

School of Human Movement

Charles Sturt University

amendham@csu.edu.au

Dr Rob Duffield

Principal Supervisor

Ph 63384939

School of Human Movement

Charles Sturt University

rduffield@csu.edu.au

Dr Aaron Coutts

Supervisor

Ph 0427 652 815

Leisure, Sport & Tourism

University of Technology Sydney

[aaron.coutts@uts.edu.au](mailto:aaron.coutts@uts.edu.au)

**Study Title:**

**The effects of 8 weeks aerobic or modified football training on skeletal muscle and biochemistry markers of mitochondrial functioning, systemic inflammation and glucose regulation.**

**Purpose of the Study**

The purpose of this study is to determine the chronic effects of a generic gym based exercise mode (cycle) in comparison to modified touch football on markers and signalling processes associated with the development of type II diabetes (T2DM) and cardiovascular disease (CVD). Additionally, the current study will assess changes involving, mitochondrial biogenesis, body composition, skeletal muscle glucose uptake, anti- and pro-inflammatory processes, and risk markers associated with T2DM and CVD.

**Study Overview and Timeline**

Participants will be required to attend **two** testing session prior to the commencement of exercise training, and **two** testing sessions after the 8 week intervention period. An outline of the testing sessions and training program is provided in the below sections.

- Information Seminar → 3^rd^ October
- Pre-intervention Testing Session → 10^th^ October– 23^th^ October
- 8 Week Training Program → 24^th^ October – 16^th^ December
- Post-intervention Testing Session → 16^th^ – 23^th^ December

**Testing Session 1 (45 min)**

In preparation;

- Complete the pre-exercise questionnaire (ESSA) and MOS SF-36 questionnaire
- No alcohol consumption in the prior 24 hr.
- Wear comfortable modest clothing that is suitable for exercise

Blood pressure and anthropometry

Firstly, participants will have their resting systolic and diastolic blood pressure measured and recorded with a blood pressure cuff and stethoscope. Additionally, mass, height, waist and hip girth measures will be measured to calculate BMI (mass [kg]/ height [m^2^]), and WHR.

Sub-maximal aerobic capacity testing

Participants will then have their aerobic exercise capacity assessed through the completion of a sub-maximal stationary cycling protocol. Heart rate will be recorded each minute throughout the protocol, and subjects exercised until volitional exhaustion or upon attainment of 80% age-predicted maximal heart rate (MHR). This aerobic fitness test will familiarise participants to the aerobic cycling protocols and will identify an initial starting point for week one of training.

Sub-maximal strength testing

Participants will then have their upper- and lower-body muscular strength assessed through the completion of a sub-maximal test protocol, in which participants will attempt ascending resistances until 3 repetitions cannot be completed.

Breakfast

At the conclusion of the aerobic exercise testing procedure, participants will be provided with a complimentary breakfast which will include cereal, toast, fruit, juice, yoghurt, and tea or coffee.

**Testing Session 2 (2.5 hours)**

In Preparation;

- Complete a 10 hr overnight fast (water only) prior to arriving at testing;
- No alcohol consumption in the prior 24 hr;
- Wear comfortable modest clothing that is suitable for exercise
- Ensure that all clothing is free from any metal accessories (i.e. zippers, belt buckles) which will disrupt the DXA scan images.

Muscle Biopsy Procedure

Upon arrival at the laboratory, a physician who has performed over 100 biopsy extraction procedures will prepare a site on the anterior thigh surface (~15cm above the kneecap) for the collection of a muscle biopsy. A local anaesthetic will be administered to the site (please inform the Chief Investigator or physician if you are allergic to anaesthetics), and once numb, a 5mm incision will be made with a scalpel. A special biopsy needle will then be inserted into the incision site and a small piece of muscle (about the size of a split pea) will be surgically removed. After collection, the site will be cleaned, sterilized, wound dressings and ice applied. As will be explained later, participants may experience some discomfort during and some soreness following muscle biopsy procedure.

Resting blood measures and Oral Glucose Tolerance Test (OGTT)

Participants will undergo an OGTT which is a commonly performed test used in assessing diabetes status (normal, pre-diabetes, diabetes). Participants will have an in-dwelling catheter inserted into a forearm vein and a resting blood sample will be collected for the measurement of fasting glucose, fasting insulin, cholesterol and inflammatory markers (IL-1ra, IL-1β, CRP, IL-6 and TNF-α). After the resting blood sample a 300 ml glucose solution will be consumed and blood samples (for measurement of C-peptide, insulin and glucose) will be collected from the catheter at 30 minute intervals until a 2 hours sample is obtained. During this 2 hour period the participants will have a DXA scan; reading material (newpapers, magazines) and a television will also be available for entertainment. The catheter will then be removed and the site will be cleaned and wound dressings applied.

DXA Scan Procedure

Whilst participants are seated during the 2 hour OGTT procedure they will be required to have a whole-body DXA scan which will take approximately 10 minutes. Participants will be assisted onto a scanning bed, and a very low-dose x-ray will be used to identify the amount of fat, muscle, and bone tissue that participants have in their torso, and upper and lower limbs. Ionising radiation will be used as part of the DXA scan. Each participant will receive approximately 2 microSieverts on two occasions (pre and post exercise training) for a total body composition scan. To provide a comparison with other radiation sources and procedures a normal chest or dental x-ray typically exposes patients to approximately 50 microSieverts. As part of everyday living, everyone is exposed to naturally occurring background radiation and receives a dose of about 2000 microSieverts each year. Participants will receive a total exposure of 4 microSieverts from the proposed DXA procedures over the duration of the study. At this dose level, no harmful effects of radiation have been demonstrated as any effect is too small to measure. According to the Australia Radiation Protection and Nuclear Safety Agency the level of risk in this project is considered minimal and is equivalent to Risk Category I (<1 ; 100,000).

**Participant Exclusion (cumulative radiation-related illness):** If you have been exposed to the more than 1000 microSieverts (over and above the natural background) within the last 12 months (International Committee of Radiological Protection). This is equivalent to approximately 20 dental or chest X-rays. If you have been exposed to this high dose of radiation please inform the chief investigator or supervisors if you have any questions or concerns involving the matter.

Breakfast

At the conclusion of the OGTT procedure, participants will be provided with a complimentary breakfast which will include cereal, toast, fruit, juice, yoghurt, and tea or coffee.

**Exercise Training Programs and Control Condition**

- **Aerobic training group** –3 days/wk
- **Touch football training group** – 3 days/wk
- It is expected that training days will be Monday, Wednesday and Fridays
- Heart rate monitors will be worn during all exercise sessions to quantify load and intensity.
- GPS units will be worn by the football group to identify movement patterns, speed and distance.
- **Control condition** - no exercise training and maintain ‘normal’ lifestyle throughout the 8 week period; participants will receive supervised exercise training following completion of the 8 week intervention period.

Aerobic (cycling) Training Program

The aerobic exercise program will involve participants performing continuous a cycling exercise on stationary ergometers for a total of 3 sessions per week. During the 8 weeks, exercise duration will increase from 40 min (weeks 1-2), 45 min (weeks 3-5) and to 50 min (weeks 6-8) with bike resistance and RPM manipulated to evoke a heart rate range of 80-85 of maximal heart rate. In each training session pedalling resistance, rpm and heart rate will be recorded at 5 minute intervals and at the conclusion an overall RPE will be obtained.

Modified Football Training Program

The modified football program will involve participants exercising for 3 sessions per week of touch football games. Additionally, during one training session per week GPS units will be utilised to identify any improvements in velocity and total distance covered throughout the exercise program. Each training session was comprises of four quarters, interspersed by 2-min passive recovery periods. Total session duration for SSG training incorporates exercise duration and 3x2 min passive recovery periods, hence total session durations were 46 min (weeks 1-2), 51 min (weeks 3-5) and 56 min (weeks 6-8). During the 8 weeks, training load progressively increased via manipulation of session duration and field size (Weeks 1-3 (25m; 40m); weeks 4-6 (35; 50 m); weeks 7-8 (40 m; 60m), including consistent game rules, verbal feedback and player numbers at 5 v 5 or 6 v 6 (depending on participant availability). At the conclusion of each session an RPE will be collected from participants.

Control Condition

The control condition will involve participants continuing their sedentary life and normal dietary and nutritional patterns for the 8 week intervention period. Participants will be provided with a dietary journal and a physical activity journal in which they will be required to document any dietary changes or physical activity patterns. Following the 8 week intervention period, participants within the control group will be invited to attend the CSU gymnasium to commence a specifically tailored exercise training program.

**Participant exclusion criteria**

***** This study is a follow-up training study which is to be based off results and findings from a recently completed acute study (single exercise session). Participants in the acute study were Caucasian males, and to compare results this training study must only involve a Caucasian population.

- This includes more than one moderate to vigorous organised exercise session per week;
- Any known diagnosed stable or unstable cardiovascular or heart disease;
- Persons with a previous diagnosis of diabetes or currently being tested for diabetes;
- Current smokers (including social smokers) or smokers quitting <12 months ago;
- Persons with a body mass index above 35.0 kg/m^2^;
- Persons with any current illnesses such as the flu, hepatitis, etc;
- Persons being treated for dental disease;
- Persons with chronic fatigue, respiratory disease, or severe asthma;
- Persons with chronic orthopaedic limitations potentially affected by exercise procedures.
- If you have been exposed to the more than 1000 microSieverts (over and above the natural background) within the last 12 months (International Committee of Radiological Protection).

**Following express permission, your general practitioner may be contacted to ensure you are free from known disease*.***

***Participant identification procedures***

- No photographs or video footage will be taken without written permission from the participant (name, signed and dated).
- If participants provide permission-video recording and/or photography may occur during the data collection, exercise training protocols, or testing procedures.
- Photos or video footage will be used to capture the methodology, training environment, and document evidence of study procedures and protocols.
- Care will be taken to photograph or videotape the person without a shot of their head in the footage/photo.
- Photo or video footage revealing participant identity, may be discarded or their identity may be blocked out through a visual block (i.e. blurring, black patch, etc).
- Photos or video footage will not be used in any published material, and participant names will not be associated with the video recordings.
- All results and data obtained throughout this study will be kept confidential, only the Chief Investigator and Research Supervisor will oversee data collection and collation.
- If a situation arises where data is to be viewed by other colleagues within the School of Human Movement Studies, participant confidentiality and anonymity will be maintained at all times.

***Participant Benefits***

Exercise participants will receive a comprehensive amount of information regarding numerous aspects of their personal health, including changes in:

- conventional and novel cardiovascular disease and diabetes screening markers;
- diabetes status (i.e. normal, pre-diabetic, or diabetic) and metabolic function;
- functional musculoskeletal capacity; increased upper- and lower-body strength;
- functional cardiovascular capacity; lower heart rates for same pedalling resistance;
- bodily weight and girths due to the exercise training;
- whole-body muscle mass, fat mass, and bone mineral content;
- free exercise training

***Participant Risks***

Participants that become involved in this study may potentially experience some risk from their involvement in some of the outlined study testing and training procedures. As such, participants need to read the below list so that they are aware of these risks prior to providing Informed Consent and becoming involved in these study procedures.

- All participants will be monitored for soreness, and will be guided through procedures which may alleviate any persisting discomfort.
- Participants may experience particular discomfort to areas of previous injury, and should consider their involvement in the study if a past injury is likely to reoccur.
- Participants may experience some discomfort during the administration of the local anaesthetic and (despite apparent analgesia) in the collection of the muscle biopsy.
- The muscle biopsy procedure is likely to be an uncomfortable experience, and soreness at the biopsy site may be experienced up to 48 hours post collection.
- Blood collection is likely to be an uncomfortable experience, and all efforts will be directed to ensuring the comfort of the participant during the procedure.
- All care will be taken to ensure a comfortable training environment is provided (room lighting, temperature, cleanliness, equipment safety, etc.), and care will be taken to avoid any potential hazards.
- The DXA scan procedure involves ionising radiation, and participants should be aware of this, and inform the Chief Investigator and/or supervisor of other recent scans (chest scan, dental scan, bone density scan, MRI, CT, DXA, etc) which may have been conducted recently.
- A separate research application form has been submitted to the CSU Radiation Safety Committee for the use of DXA in this study, and if these procedures are deemed to be safe and of minimal risk to participants, these procedures will be approved.

**Participant Care and Follow-up**

After all muscle biopsy and testing procedures, the chief investigator will ensure a follow up phone call to all participants within the 24 hour period. This will ensure all participants have appropriate follow-up care. Additionally, all participants will receive a written report and verbal feedback from the Chief Investigator regarding their testing measures. The written report will include comparisons to normal healthy reference values, and target values for those values that are outside the normal range. If testing procedures reveal an abnormality, or an imminent or new disease condition, the Chief Investigator will inform the respective participant in person, and will further notify their general practitioner of these findings and provide information regarding the suspected abnormality or disease condition

**Data Collection and Research Publications**

The data collected during medical procedures, exercise testing procedures, or exercise training procedures may potentially be utilised for multiple publication in a health/exercise science Journal.

Note: Charles Sturt University’s Ethics in Human Research Committee has approved this project. If you have any complaints or reservations about the ethical conduct of this project, you may contact the Committee treatment through the executive Officer:

The Executive Officer, Ethics in Human Research Committee,

Academic Secretariat, Charles Sturt University,

Private Mail Bag 29, Bathurst, NSW, 2795.

Tel: (02) 6338 4628, Fax: (02) 6338 4194.

Any issues you raise will be treated in confidence and investigated fully and you will be informed of the outcome.
