# Supplementary material for: Rugby-Specific Small-Sided Games Training Is an Effective Alternative to Stationary Cycling at Reducing Clinical Risk Factors Associated with the Development of Type 2 Diabetes: A Randomized, Controlled Trial
Source: PLoS One. 2015 Jun 1;10(6):e0127548. doi: 10.1371/journal.pone.0127548 (PMC4452519; doi:10.1371/journal.pone.0127548)
Supplement: S4 File — Tables reporting raw data for all primary and secondary variables assessed. (PDF) [file pone.0127548.s004.pdf]

Football			Cycling			Control		
**Subject**	**Age**	**Height (m)**	**Subject**	**Age**	**Height (m)**	**Subject**	**Age**	**Height (m)**
			11	49.0	1.8	22	53.0	1.8
1	44.0	1.8	12	49.0	1.7	23	54.0	1.8
2	54.0	1.6	13	39.0	1.8	24	55.0	1.7
3	50.0	1.7	14	48.0	1.8	25	52.0	1.8
4	53.0	1.8	15	61.0	1.7	26	34.0	1.8
5	37.0	1.7	16	45.0	1.8	27	45.0	1.8
6	45	1.8	17	43.0	1.8	28	40.0	1.7
7	57	1.8	18	51.0	1.695	29	57.0	1.8
8	47.0	1.9	19	56.0	1.79	30	47.0	1.78
9	41.0	1.8	20	46.0	1.79	31	52.0	1.9
10	40.0	1.8	21	58.0	1.765	32	52.0	1.775
M	46.8	1.76		49.5	1.76		49.2	1.8
SD	6.60	0.08		6.61	0.04		7.03	0.06
SEM	2.09	0.02		1.99	0.01		2.12	0.02

Mass (kg)

Football			Cycling			Control		
**Subject**	**Pre**	**Post**	**Subject**	**Pre**	**Post**	**Subject**	**Pre**	**Post**
			11	82.24	82.24	22	101.9	101.28
1	98.74	97.46	12	91.18	91.76	23	84.24	84.42
2	78.7	80.4	13	97.3	95.9	24	67.8	68.7
3	70.4	70.3	14	116.5	116.3	25	84.6	85.9
4	80.8	79.7	15	93.6	92.9	26	95.7	98.6
5	75.86	74.5	16	95.6	95.9	27	92.4	91.6
6	83.9	83.5	17	96.4	97.1	28	89.6	89.5
7	69.3	70.6	18	71.3	69.9	29	108.4	108.8
8	104.2	104.0	19	75.8	75.2	30	99.4	96.4
9	96.48	95.6	20	91.5	90.63	31	91.42	92.24
10	104.9	105.4	21	81.6	80.32	32	103.1	103.08
M	86.3	86.1		90.3	89.8		92.6	92.8
SD	13.6	13.4		12.3	12.6		11.2	10.9
SEM	4.3	4.2		3.7	3.8		3.4	3.3

BMI

Football			Cycling			Control		
**Subject**	**Pre**	**Post**	**Subject**	**Pre**	**Post**	**Subject**	**Pre**	**Post**
			11	26.07	26.07	22	33.27	33.07
1	29.81	29.42	12	32.11	32.32	23	27.04	27.10
2	29.48	30.11	13	28.75	28.33	24	24.91	25.23
3	24.22	24.18	14	36.60	36.55	25	26.10	26.51
4	25.21	24.87	15	31.05	30.82	26	30.20	31.11
5	27.33	26.83	16	31.05	31.15	27	29.31	29.06
6	26.78	26.65	17	30.76	30.99	28	29.59	29.55
7	22.63	23.05	18	24.81	24.34	29	33.46	33.59
8	29.79	29.75	19	23.66	23.48	30	31.37	30.42
9	29.32	29.06	20	28.55	28.29	31	25.06	25.28
10	31.67	31.82	21	26.21	25.78	32	32.72	32.72
M	27.62	27.57		29.06	28.92		29.47	29.42
SD	2.89	2.90		3.76	3.90		3.20	3.07
SEM	0.92	0.92		1.13	1.17		0.97	0.92

W/G (cm)

Football Subject	Pre	Post	Cycling Subject	Pre	Post	Control Subject	Pre	Post
			11	88.5	87	22	100	100
1	93.5	93.5	12	108.5	105.5	23	89	89
2	98	98	13	104	104.5	24	84	84.5
3	98.5	86	14	113	111	25	89	90
4	88	86	15	98	98	26	102.5	102
5	93	85.5	16	95	94	27	103	96
6	92	89	17	95	101	28	102	100.5
7	82	82	18	84	81.5	29	112	112
8	98.5	97	19	92	87	30	106	107
9	100	99	20	95.5	92	31	95	95
10	105	105	21	93	90	32	107	107
0.064			0.151			0.401		
M	94.85	92.10		96.95	95.59		99.05	98.45
SD	6.60	7.49		8.55	9.18		8.72	8.50
SEM	2.09	2.37		2.58	2.77		2.63	2.56

H/G (cm)

Football Subject	Pre	Post	Cycling Subject	Pre	Post	Control Subject	Pre	Post
			11	96	96	22	108	108
1	105	104.5	12	101	98	23	98.5	99.5
2	100	98	13	103	103	24	94	94.5
3	89.5	88.5	14	107.5	103.5	25	92	92
4	99	97	15	106	106	26	104.5	104
5	96	93	16	110	107	27	101	100
6	95	95	17	102	100	28	103.5	103.5
7	92.5	92.5	18	94	92	29	112	112
8	105.5	103.5	19	97	96	30	107	108
9	106	103	20	100	99	31	103	103
10	109.5	108	21	100	100	32	112	112
M	99.80	98.30		101.50	100.05		103.23	103.32
SD	6.58	6.25		4.92	4.55		6.57	6.53
SEM	2.08	1.98		1.48	1.37		1.98	1.97

WHR

Football Subject	Pre	Post	Cycling Subject	Pre	Post	Control Subject	Pre	Post
			11	0.92	0.91	22	0.93	0.93
1	0.89	0.89	12	1.07	1.08	23	0.90	0.89
2	0.98	1.00	13	1.01	1.01	24	0.89	0.89
3	1.10	0.97	14	1.05	1.07	25	0.97	0.98
4	0.89	0.89	15	0.92	0.92	26	0.98	0.98
5	0.97	0.92	16	0.86	0.88	27	1.02	0.96
6	0.97	0.94	17	0.93	1.01	28	0.99	0.97
7	0.89	0.89	18	0.89	0.89	29	1.00	1.00
8	0.93	0.94	19	0.95	0.91	30	0.99	0.99
9	0.94	0.96	20	0.96	0.93	31	0.92	0.92
10	0.96	0.97	21	0.93	0.90	32	0.96	0.96
M	0.95	0.94		0.95	0.95		0.96	0.95
SD	0.06	0.04		0.06	0.07		0.04	0.04
SEM	0.02	0.01		0.02	0.02		0.01	0.01

Total Chol

Football Subject	PRE	POST	Cycling Subject	PRE	POST	Control Subject	PRE	POST
1.0	4.9	4.9	11	5.0	4.0	22	4.5	5.2
2.0	6.0	5.8	12	4.8	5.2	23	4.8	4.6
3.0	4.6	4.1	13	5.7	6.0	24	5.4	6.2
4.0	5.6	4.5	14	6.2	5.7	25	5.2	4.6
5.0	4.4	4.3	15	4.8	5.0	26	5.8	5.0
6.0	7.3	6.6	16	6.2	5.6	27	4.0	4.2
7.0	5.5	5.4	17	4.3	4.6	28	3.1	3.9
8.0	4.9	5.2	18	4.0	4.3	29	5.2	5.2
9.0	6.7	6.6	19	6.4	6.4	30	5.5	5.2
10.0	4.4	4.7	20	5.9	5.5	31	5.7	5.4
			21	5.1	4.2	32	6.8	6.2
M	5.4	5.2		5.3	5.1		5.1	5.1
SD	1.0	0.9		0.8	0.8		1.0	0.7
SEM	0.3	0.3		0.2	0.2		0.3	0.2

LDL

Football Subject	PRE	POST	Cycling Subject	PRE	POST	Control Subject	PRE	POST
			11	3.3	2.3	22	2.7	3.7
1.0	3.2	3.1	12	3.0	3.2	23	2.8	2.5
2.0	3.6	3.7	13	3.6	4.2	24	4.1	3.4
3.0	2.8	2.3	14	3.9	3.7	25	2.8	2.5
4.0	3.8	2.8	15	3.4	3.2	26	4.0	3.3
5.0	2.6	2.4	16	4.4	3.8	27	2.5	2.7
6.0	5.2	4.2	17	2.1	2.2	28	2.1	2.4
7.0	4.0	3.8	18	2.4	2.5	29	3.5	3.4
8.0	3.3	3.5	19	4.2	4.1	30	3.2	3.8
9.0	5.1	4.7	20	4.3	3.8	31	3.7	3.6
10.0	2.2	2.4	21	3.3	2.5	32	4.8	4.1
M	3.6	3.3		3.4	3.2		3.3	3.2
SD	1.0	0.8		0.7	0.7		0.8	0.6
SEM	0.3	0.3		0.2	0.2		0.2	0.2

HDL

Football Subject	PRE	POST	Cycling Subject	PRE	POST	Control Subject	PRE	POST
			11	1.2	1.2	22	0.8	0.8
1.0	1.2	1.3	12	1.3	1.5	23	1.7	1.8
2.0	1.0	1.1	13	1.2	1.2	24	1.7	1.7
3.0	1.2	1.1	14	1.0	0.8	25	2.0	1.5
4.0	1.5	1.3	15	1.0	1.0	26	1.3	1.3
5.0	1.4	1.5	16	1.3	1.3	27	1.1	1.2
6.0	1.0	1.0	17	1.7	1.9	28	0.8	0.7
7.0	1.0	1.1	18	1.5	1.6	29	1.1	1.1
8.0	1.1	1.2	19	1.6	1.5	30	1.0	1.0
9.0	1.0	1.0	20	1.2	1.2	31	1.6	1.3
10.0	1.6	1.8	21	1.2	1.3	32	1.5	1.4
M	1.2	1.2		1.3	1.3		1.3	1.2
SD	0.2	0.3		0.2	0.3		0.4	0.4
SEM	0.1	0.1		0.1	0.1		0.1	0.1

TRIGS

Football Subject	PRE	POST	Cycling Subject	PRE	POST	Control Subject	PRE	POST
			11	1.1	1.2	22	1.6	2.3
1.0	1.0	1.2	12	1.3	1.1	23	0.8	0.8
2.0	3.1	2.1	13	1.9	1.4	24	0.7	0.6
3.0	1.3	1.6	14	2.8	2.5	25	0.8	1.3
4.0	0.8	0.7	15	1.0	1.8	26	1.0	0.9
5.0	1.0	1.0	16	1.0	1.0	27	0.8	0.6
6.0	2.3	3.0	17	1.1	1.1	28	0.5	2.0
7.0	1.0	1.0	18	0.3	0.4	29	1.3	1.5
8.0	1.2	1.1	19	1.4	1.9	30	2.9	1.1
9.0	1.3	2.0	20	1.1	1.0	31	1.0	1.2
10.0	1.5	1.1	21	1.3	0.9	32	1.3	1.5
M	1.4	1.5		1.3	1.3		1.1	1.3
SD	0.7	0.7		0.6	0.6		0.7	0.5
SEM	0.2	0.2		0.2	0.2		0.2	0.2

Hazard Ratio

Football Subject	PRE	POST	Cycling Subject	PRE	POST	Control Subject	PRE	POST
			11	4.2	3.5	22	6.2	5.8
1.0	3.9	3.9	12	3.8	3.6	23	2.9	2.6
2.0	5.9	5.3	13	4.7	5.0	24	3.2	3.6
3.0	3.9	3.8	14	6.5	7.0	25	2.6	3.1
4.0	3.8	3.3	15	4.7	4.9	26	4.4	4.0
5.0	3.3	2.9	16	4.8	4.2	27	3.6	3.6
6.0	7.0	6.8	17	2.6	2.5	28	4.0	6.0
7.0	5.5	5.0	18	2.7	2.6	29	4.7	5.0
8.0	4.5	4.4	19	4.0	4.3	30	5.8	5.3
9.0	6.5	6.8	20	5.1	4.4	31	3.6	4.2
10.0	2.8	2.6	21	4.2	3.2	32	4.7	4.4
M	4.7	4.5		4.3	4.2		3.9	4.2
SD	1.4	1.5		1.1	1.3		1.0	1.1
SEM	0.5	0.5		0.3	0.4		0.3	0.3

Gluocse

Football Subject	PRE	POST	Cycling Subject	PRE	POST	Control Subject	PRE	POST
			11	5.2	4.1	22	2.9	3.7
1.0	3.8	4.5	12	5.0	5.4	23	4.7	5.2
2.0	5.4	4.3	13	5.3	5.1	24	3.8	4.0
3.0	5.5	5.0	14	4.7	4.8	25	4.8	5.4
4.0	3.7	3.7	15	6.4	6.1	26	6.2	6.0
5.0	4.3	3.3	16	4.6	4.4	27	5.5	4.2
6.0	5.0	4.9	17	5.3	5.3	28	4.7	3.9
7.0	4.4	4.1	18	3.7	4.2	29	5.2	5.8
8.0	4.9	4.8	19	3.9	4.0	30	4.4	4.1
9.0	4.7	4.6	20	4.6	3.9	31	5.4	5.2
10.0	5.3	4.8	21	4.2	4.7	32	5.2	5.3
M	4.7	4.4		4.8	4.7		4.8	4.8
SD	0.6	0.6		0.7	0.7		0.9	0.8
SEM	0.2	0.2		0.2	0.2		0.3	0.3

Insulin

Football			Cycling			Control		
Subject	PRE	POST	Subject	PRE	POST	Subject	PRE	POST
			11	2.9	2.0	22	5.6	6.0
1.0	4.5	4.1	12	9.1	11.3	23	2.0	2.0
2.0	25.6	12.5	13	9.6	7.6	24	2.0	2.1
3.0	5.7	3.5	14	25.3	20.2	25	2.0	4.8
4.0	2.0	2.0	15	21.7	21.1	26	17.1	21.3
5.0	3.2	3.4	16	8.8	9.4	27	12.7	8.7
6.0	5.0	3.4	17	7.7	5.8	28	14.2	16.9
7.0	4.8	3.3	18	2.0	2.0	29	19.8	27.3
8.0	4.0	12.1	19	2.2	2.0	30	11.3	9.3
9.0	11.1	5.2	20	2.0	2.0	31	2.0	4.0
10.0	9.2	4.1	21	2.3	2.1	32	8.0	8.4
M	7.5	5.3		8.5	7.8		8.8	10.1
SD	6.9	3.8		8.1	7.2		6.6	8.3
SEM	2.2	1.2		2.4	2.2		2.0	2.5

HbA1c

Football			Cycling			Control		
Subject	PRE	POST	Subject	PRE	POST	Subject	PRE	POST
			11	5.2	5.0	22	5.3	5.3
1.0	5.9	5.5	12	6.3	6.0	23	6.1	6.0
2.0	6.2	5.7	13	5.8	5.6	24	5.6	5.2
3.0	6.5	6.1	14	5.3	5.1	25	5.8	5.6
4.0	5.4	5.4	15	7.4	6.3	26	6.2	6.0
5.0	5.1	4.9	16	5.3	5.3	27	5.5	5.5
6.0	5.8	5.6	17	5.5	5.5	28	5.5	5.6
7.0	5.9	5.5	18	5.4	5.2	29	5.3	5.8
8.0	5.2	5.0	19	5.7	5.6	30	4.8	4.7
9.0	5.6	5.3	20	5.0	4.8	31	5.5	5.6
10.0	5.4	5.3	21	5.4	5.1	32	5.3	5.4
M	5.7	5.4		5.7	5.4		5.5	5.5
SD	0.4	0.3		0.7	0.4		0.4	0.4
SEM	0.1	0.1		0.2	0.1		0.1	0.1

HOMA-IS

Football			Cycling			Control		
Subject	PRE	POST	Subject	PRE	POST	Subject	PRE	POST
			11	0.7	0.4	22	0.7	1.0
1.0	0.8	0.8	12	2.0	2.7	23	0.4	0.5
2.0	6.1	2.4	13	2.3	1.7	24	0.3	0.4
3.0	1.4	0.8	14	5.2	4.3	25	0.4	1.1
4.0	0.3	0.3	15	6.1	5.7	26	4.7	5.7
5.0	0.6	0.5	16	1.8	1.8	27	3.1	1.6
6.0	1.1	0.7	17	1.8	1.4	28	3.0	2.9
7.0	0.9	0.6	18	0.3	0.4	29	4.5	7.0
8.0	0.9	2.6	19	0.4	0.4	30	2.2	1.7
9.0	2.3	1.1	20	0.4	0.3	31	0.5	0.9
10.0	2.2	0.9	21	0.4	0.4	32	1.9	2.0
M	1.7	1.1		1.9	1.8		2.0	2.3
SD	1.7	0.8		2.0	1.8		1.7	2.2
SEM	0.5	0.2		0.6	0.5		0.5	0.7

Pre-Training

Football

Subjects	TB-LM (g)	TB-FM (g)	TB-FM (%)	Abdominal FM (g)
1	68213	26402	26.9	2474
2	54776	23359	28.7	2748
3	51853	15126	21.6	2109
4	55237	21886	26.9	1498
5	51462	19641	26.3	1854
6	59450	21367	25.3	2527
7	48323	17563	25.5	1542
8	70259	30318	29.0	2776
9	65746	27752	28.6	2946
10	67385	34877	32.8	3953
Mean	59270	23829	27.2	2443
SD	8034	6044	2.9	738
SEM	2540.416	1911.304	0.931	233.532

Post-Training

Football

Subjects	TB-LM (g)	TB-FM (g)	TB-FM (%)	Abdominal FM (g)
1	69188	25628	26.05	2479
2	53957	22789	28.49	2642
3	54066	14524	20.29	1919
4	56586	20342	25.10	1468
5	54975	17064	22.60	1625
6	59553	21799	25.68	2493
7	49131	17953	25.62	1649
8	70586	29400	28.30	2804
9	67569	26867	27.39	2994
10	68221	34339	32.24	3849
	60383	23071	26.18	2392
	7795	6078	3.29	742
	2465.000	1921.938	1.040	234.594

Cycling (Pre-Training)

Subjects	TB-LM (g)	TB-FM (g)	TB-FM (%)	Abdominal FM (g)
11	57114	19406	24.1	1780
12	57991	30057	33.0	3741
13	64375	29209	30.0	3140
14	67947	44731	38.3	4901
15	58388	32544	34.5	3358
16	68952	23647	24.5	2106
17	66788	27680	28.1	3047
18	54616	12751	18.0	1047
19	48079	25287	33.0	2251
20	58321	30933	33.3	2679
21	60883	17463	21.4	1851
Mean	60314	26701	28.9	2718
SD	6301	8603	6.3	1074
SEM	1899.967	2593.925	1.886	323.698

Cycling (Post-Training)

Subjects	TB-LM (g)	TB-FM (g)	TB-FM (%)	Abdominal FM (g)
11	59435	17807	21.89	1636
12	60158	29803	32.05	3835
13	65678	27838	28.65	3082
14	70737	42721	36.39	4724
15	58485	32261	34.24	3088
16	68871	24119	24.88	2194
17	65875	28038	28.66	2789
18	53203	12514	18.12	827
19	49784	24721	31.78	2160
20	58722	29828	32.33	2500
21	60836	15843	19.84	1619
	61071	25954	28.08	2587
	6353	8442	6.09	1093
	1915.598	2545.500	1.836	329.539

Pre-Training

Control

Subjects	TB-LM (g)	TB-FM (g)	TB-FM (%)	Abdominal FM (g)
22	68740	31615	30.3	2612
23	63454	16848	20.1	1502
24	48021	17549	25.7	1755
25	61510	18110	21.7	1307
26	63686	30261	31.0	3089
27	59997	28532	30.8	2799
28	60506	26895	29.8	2988
29	65656	41045	37.0	4226
30	57965	40986	40.0	4044
31	71452	14419	16.0	1627
32	67712	32055	31.0	3198
Mean	62609	27120	28.5	2650
SD	6323	9393	7.1	1001
SEM	1906.487	2832.048	2.146	301.919

Post-Training

Control

Subjects	TB-LM (g)	TB-FM (g)	TB-FM (%)	Abdominal FM (g)
22	65840	31415	30.98	2556
23	64882	16728	19.63	1579
24	46843	19313	28.02	1736
25	62849	20117	23.21	1576
26	65644	32342	31.87	3385
27	58999	28834	31.38	2916
28	57752	28927	32.26	3052
29	65656	41045	36.96	4226
30	54179	40143	41.06	3956
31	70667	17030	18.55	1705
32	66756	31091	30.62	3504
	61824	27908	29.51	2745
	6825	8626	6.84	981
	2057.731	2600.978	2.063	295.890

PRE Training
Football

Subject	est Duration (mi	Last WL (Watts)	$VO_{2submax}$ kg/ml/min
1	8.08	250	26.2
2	5.42	175	22.73
3	5.87	175	19.7
4	7.22	225	30.8
5	7.52	225	28.1
6	4.75	150	20.1
7	7.08	225	25.6
8	4.60	150	21.3
9	9.23	275	26.2
10	6.95	200	26.4
M	6.7	205.0	24.7
SD	1.5	42.2	3.6
SEM	0.5	13.3	1.1

POST Training
Football

Subject	est Duration (mi	Last WL (Watts)	$VO_{2submax}$ kg/ml/min
1	9.72	275	30.33
2	6.62	200	24.6
3	9.57	275	29.05
4	7.93	225	33
5	8.25	250	29.76
6	8.22	250	32.28
7	8.35	250	27.65
8	5.53	175	24.14
9	9.47	275	28.36
10	8.97	250.0	30.17
	8.3	242.5	28.9
	1.3	33.4	2.9
	0.4	10.6	0.9

Cycling (PRE)

Subject		Last WL (Watts)	$VO_{2submax}$ kg/ml/min
11	4.42	150	15
12	7.32	225	28.1
13	7.78	225	24.6
14	8.85	250	30.2
15	5.72	175	19.3
16	8.27	250	25.05
17	7.90	225	25.3
18	5.87	175	24.5
19	5.87	175	22.5
20	7.77	225	25.4
21	6.83	200.0	26.1
M	7.0	206.8	24.2
SD	1.3	33.7	4.1
SEM	0.4	10.2	1.2

Cycling (POST)

Subject		Last WL (Watts)	$VO_{2submax}$ kg/ml/min
11	8.15	250	27.9
12	8.67	250	32
13	9.10	275	28.2
14	11.95	325	35
15	6.15	200	22.4
16	8.95	275	26
17	9.18	275	25.9
18	8.13	250	27.5
19	7.42	225	28.3
20	8.08	250.0	30.1
21	8.48	250.0	26.4
	8.6	256.8	28.1
	1.4	31.8	3.3
	0.4	9.6	1.0

PRE Training
Control

Subject		Last WL (Watts)	$VO_{2submax}$ kg/ml/min
22	7.52	225	28.1
23	7.08	225	28.8
24	7.53	225	35.9
25	7.67	225	24.8
26	7.08	225	25.1
27	8.33	250	28.8
28	8.95	250	28.3
29	4.82	150	15.7
30	8.28	250	23.3
31	9.20	275	27.7
32	6.72	200	21.2
M	7.5	227.3	26.5
SD	1.2	32.5	5.4
SEM	0.4	9.8	1.6

POST Training
Control

Subject		Last WL (Watts)	$VO_{2submax}$ kg/ml/min
22	7.52	225	28.1
23	6.08	200	24.1
24	7.53	225	32.0
25	7.85	225	25.8
26	8.08	250	24.8
27	8.33	250	28.8
28	9.45	275	27.6
29	4.82	150	14.6
30	8.58	250	22.8
31	9.20	275	27.0
32	6.72	200.0	21.2
	7.6	229.5	25.7
	1.4	36.8	5.2
	0.4	11.1	1.4

GLUCOSE - AUC

SSG

Subject	Pre-30	30-60	60-90	90-120	SUM
PRE-Training					
1	2.4	2.8	2.5	**2.1**	9.8
2	3.4	4.5	4.9	**4.8**	17.6
3	3.3	4.4	4.6	**3.4**	15.7
4	2.3	2.3	2.2	**2.6**	9.5
5	3.4	4.3	3.9	**2.9**	14.5
6	2.8	2.9	2.5	**1.8**	10.0
7	2.5	2.4	1.8	**1.6**	8.3
8	3.0	3.9	3.9	**2.9**	13.7
9	3.2	4.1	3.7	**3.4**	14.5
10	3.5	4.2	3.4	**2.6**	13.6
**M**	3.0	3.6	3.3	**2.8**	12.7
**SD**	0.4	0.9	1.1	**0.9**	3.1
**SEM**	0.1	0.3	0.3	0.3	1.0

Cycling

Subject	Pre-30	30-60	60-90	90-120	SUM
11	3.7	4.9	3.5	**2.3**	14.3
12	3.5	5.2	5.5	**5.1**	19.3
13	3.9	5.0	5.0	**4.6**	18.5
14	3.3	4.1	3.9	**3.4**	14.6
15	4.2	5.6	5.9	**4.6**	20.3
16	2.9	3.0	2.1	**2.0**	9.9
17	3.4	3.6	2.7	**2.0**	11.7
18	2.7	2.9	2.3	**2.3**	10.1
19	2.8	3.2	2.8	**2.4**	11.1
20	3.2	3.7	2.9	**2.3**	12.0
21	2.9	3.7	3.3	**2.8**	12.7
**M**	3.3	4.1	3.6	**3.0**	14.1
**SD**	0.5	0.9	1.3	**1.2**	3.7
**SEM**	0.1	0.3	0.4	0.3	1.1

Control

Subject	Pre-30	30-60	60-90	90-120	SUM
22	2.5	3.6	3.2	**2.3**	11.6
23	3.2	3.5	2.5	**1.9**	11.1
24	2.8	3.7	3.6	**3.2**	13.2
25	3.1	3.8	3.2	**2.2**	12.3
26	4.2	5.5	5.4	**4.8**	19.8
27	3.6	3.4	2.3	**2.1**	11.3
28	3.0	3.2	2.8	**2.5**	11.5
29	3.5	3.8	3.1	**2.6**	13.0
30	2.5	2.6	2.5	**2.7**	10.3
31	3.4	4.7	4.3	**2.9**	15.3
32	3.3	3.9	3.6	**2.9**	13.7
**M**	3.2	3.8	3.3	**2.7**	13.0
**SD**	0.5	0.7	0.9	**0.8**	2.7
**SEM**	0.2	0.2	0.3	0.2	0.8

SSG

Subject	Pre-30	30-60	60-90	90-120	SUM
Post Training					
1	2.7	2.3	1.5	**1.4**	7.9
2	3.0	3.5	3.0	**2.6**	12.0
3	3.0	3.6	3.1	**2.0**	11.7
4	2.3	2.3	2.2	**2.6**	9.5
5	2.4	2.8	2.2	**2.1**	9.4
6	3.0	3.2	2.6	**2.0**	10.7
7	2.7	3.0	2.3	**1.7**	9.6
8	3.3	3.8	2.7	**1.9**	11.7
9	3.3	3.8	3.0	**2.3**	12.4
10	2.8	2.8	2.0	**1.9**	9.5
**M**	2.8	3.1	2.4	**2.0**	10.4
**SD**	0.3	0.6	0.5	**0.4**	1.5
**SEM**	0.1	0.2	0.1	0.1	0.4

Cycling

Subject	Pre-30	30-60	60-90	90-120	SUM
11	3.0	3.5	2.6	**1.7**	10.8
12	3.8	4.3	3.4	**3.0**	14.5
13	3.4	4.7	4.0	**3.1**	15.2
14	3.2	3.6	3.0	**2.6**	12.3
15	4.3	5.8	5.9	**5.3**	21.3
16	2.7	3.1	2.5	**1.7**	9.9
17	2.8	2.8	2.2	**1.4**	9.1
18	2.8	2.5	1.5	**1.2**	7.9
19	2.5	2.6	2.1	**1.9**	9.1
20	2.7	3.1	2.6	**2.4**	10.7
21	2.8	3.1	2.4	**1.8**	10.1
**M**	3.1	3.5	2.9	**2.4**	11.9
**SD**	0.5	1.0	1.2	**1.2**	3.9
**SEM**	0.2	0.3	0.4	0.4	1.2

Control

Subject	Pre-30	30-60	60-90	90-120	SUM
22	2.7	3.0	2.1	**1.6**	9.4
23	3.4	3.9	3.6	**2.6**	13.4
24	2.6	3.3	2.8	**2.2**	10.9
25	3.8	4.8	3.9	**2.6**	15.1
26	3.4	3.4	3.3	**2.5**	12.5
27	3.1	3.1	2.1	**2.0**	10.3
28	3.3	4.2	3.5	**3.2**	14.2
29	4.5	5.4	4.8	**4.1**	18.8
30	2.6	3.3	3.1	**2.8**	11.7
31	3.2	4.1	3.5	**2.4**	13.2
32	3.7	4.6	3.4	**2.3**	14.0
**M**	3.3	3.9	3.3	**2.6**	13.0
**SD**	0.6	0.8	0.8	**0.7**	2.6
**SEM**	0.2	0.2	0.2	0.2	0.8

INSULIN - AUC

SSG

Subject	Pre-30	30-60	60-90	90-120	SUM
	**PRE-Training**				
1	13.0	33.8	31.5	17.5	95.8
2	24.5	73.1	105.8	93.0	296.3
3	5.7	14.6	23.7	24.6	68.7
4	8.3	12.1	9.9	9.4	39.7
5	4.4	17.4	26.1	19.3	67.1
6	8.2	15.1	16.2	14.2	53.7
7	13.9	24.3	19.2	13.2	70.6
8	14.3	30.5	49.0	55.4	149.2
9	16.7	33.4	33.6	26.3	110.0
10	13.1	22.8	20.6	14.0	70.4
**M**	12.2	27.7	33.5	28.7	102.1
**SD**	5.9	17.8	27.6	26.1	75.0
**SEM**	1.9	5.6	8.7	8.3	23.7

Cycling

Subject	Pre-30	30-60	60-90	90-120	SUM
11	9.6	25.6	21.8	10.5	67.5
12	14.6	31.9	46.3	53.8	146.5
13	12.8	16.2	17.5	23.2	69.7
14	33.6	51.9	52.6	47.7	185.7
15	23.0	64.4	112.5	105.5	305.4
16	24.0	36.2	21.3	12.9	94.3
17	15.3	35.7	32.4	13.7	97.1
18	17.4	27.2	17.4	15.4	77.4
19	4.3	7.5	8.6	11.5	31.8
20	11.1	20.3	16.2	12.2	59.8
21	6.6	16.8	18.7	16.5	58.5
**M**	15.7	30.3	33.2	29.3	108.5
**SD**	8.5	16.5	29.5	29.3	78.4
**SEM**	2.6	5.0	8.9	8.8	23.6

Control

Subject	Pre-30	30-60	60-90	90-120	SUM
22	12.9	33.6	43.1	30.9	120.5
23	3.5	7.6	8.9	7.9	28.0
24	4.6	10.2	14.5	16.8	46.0
25	4.7	11.5	15.1	11.9	43.1
26	22.7	51.1	73.3	79.3	226.3
27	29.9	43.7	29.0	24.1	126.8
28	21.6	35.2	35.3	27.5	119.5
29	53.0	76.0	63.0	66.0	258.0
30	14.5	27.8	28.5	27.1	97.9
31	5.0	13.2	16.6	15.3	50.0
32	14.7	31.3	36.8	29.0	111.7
**M**	17.0	31.0	33.1	30.5	111.6
**SD**	14.7	20.8	20.5	22.3	74.1
**SEM**	4.4	6.3	6.2	6.7	22.3

SSG

Subject	Pre-30	30-60	60-90	90-120	SUM
	**Post Training**				
1	8.1	13.4	12.8	13.8	48.0
2	16.1	42.5	56.3	54.3	169.0
3	4.4	12.6	20.6	21.9	59.5
4	6.7	12.4	11.8	9.6	40.5
5	7.5	12.5	11.2	11.8	42.8
6	6.7	14.2	14.0	10.6	45.5
7	11.1	22.7	20.2	13.9	67.9
8	10.2	27.5	28.5	15.8	82.0
9	14.8	30.6	37.3	29.4	112.1
10	10.6	18.7	12.4	8.6	50.3
**M**	9.6	23.3	22.5	19.0	71.7
**SD**	3.7	14.1	14.6	13.9	40.7
**SEM**	1.2	3.2	4.6	4.4	12.9

Cycling

Subject	Pre-30	30-60	60-90	90-120	SUM
11	4.7	11.5	13.7	11.9	41.7
12	19.2	45.4	61.0	61.0	186.6
13	5.1	12.6	41.7	49.4	108.7
14	29.9	50.4	40.6	33.9	154.8
15	23.2	55.9	91.5	91.8	262.4
16	25.6	48.5	39.0	24.0	137.0
17	14.4	37.1	33.6	12.3	97.4
18	12.2	21.9	17.1	10.2	61.4
19	4.0	10.3	9.8	7.5	31.6
20	1.0	4.8	8.6	10.6	25.0
21	7.1	14.0	12.4	10.1	43.6
**M**	13.3	28.4	33.5	29.3	104.6
**SD**	9.9	19.2	25.6	27.4	75.1
**SEM**	3.0	5.8	7.7	8.3	22.7

Control

Subject	Pre-30	30-60	60-90	90-120	SUM
22	18.3	55.3	48.5	15.4	137.5
23	3.5	6.6	13.6	53.8	77.5
24	5.2	13.3	31.1	28.8	78.3
25	8.0	17.6	20.2	13.1	58.8
26	29.8	57.2	68.0	48.7	203.7
27	31.4	46.5	26.7	16.2	120.7
28	55.2	86.8	59.5	68.2	269.6
29	60.1	88.8	81.3	75.8	305.8
30	12.0	21.9	29.1	28.5	91.4
31	4.7	11.5	18.2	20.8	55.2
32	12.1	54.0	60.6	22.6	149.2
**M**	21.8	41.7	41.5	35.6	140.7
**SD**	20.1	29.6	23.0	22.3	85.2
**SEM**	6.1	8.9	6.9	6.7	25.7

Matsuda ISI

	SSG Pre	SSG Post	CYC Pre	CYC Post	CON Pre	CON Post
	9.6	13.9	10.2	19.9	8.3	7.4
	1.4	3.6	3.3	2.9	22.0	9.9
	6.4	10.7	4.6	4.6	17.2	14.7
	22.6	22.4	2.1	2.8	17.1	8.2
	10.5	17.5	1.3	1.4	1.7	2.1
	10.2	13.4	6.2	5.2	3.8	5.7
	10.8	13.0	5.8	7.5	4.0	2.3
	6.0	5.1	15.7	19.3	2.0	1.3
	4.1	6.8	20.9	24.4	5.1	5.8
	5.6	12.2	14.9	24.6	13.2	9.5
**M**	8.7	11.8	8.5	11.3	9.4	6.7
**SD**	5.8	5.7	6.7	9.6	7.4	4.2
**SEM**	1.8	1.8	2.1	3.0	2.3	1.3

NRF2

CON	pre	post
25	0.29	0.39
23	0.31	0.32
27	0.18	0.39
22	0.44	0.36
32	0.52	1.23
28	1.17	1.42
26	1.30	1.16
24	3.57	0.95
31	1.23	1.34
M	1.00	0.84
SD	1.06	0.47
SE	0.35	0.16

CYC	pre	post
19	0.33	0.35
16	0.39	0.56
15	1.37	0.54
12	0.59	0.76
14	1.13	1.97
13	1.53	1.40
17	0.56	2.42
21	1.31	1.26
11	1.41	0.97
18	1.50	2.31
20	0.86	0.59
M	1.00	1.26
SD	0.49	0.76
SE	0.15	0.24

SSG	pre	post
9	0.91	0.51
8	0.18	0.60
1	0.64	1.03
3	0.31	0.40
4	0.88	0.53
7	3.81	2.62
5	0.72	0.32
10	0.59	0.38
2	1.65	0.82
6	0.30	0.57
M	1.00	0.78
SD	1.07	0.68
SE	0.34	0.22

NRF1

CON	pre	post
25	0.34	0.77
23	0.78	0.93
27	0.65	0.38
22	0.79	0.28
32	1.78	2.20
28	0.67	1.21
26	1.22	0.54
24	0.61	0.52
31	2.17	2.00
M	1.00	0.98
SD	0.61	0.69
SE	0.20	0.23

CYC	pre	post
19	0.31	0.31
16	1.91	1.65
15	0.23	0.60
12	2.01	1.27
14	1.53	1.37
13	0.95	1.07
17	0.93	2.48
21	0.80	0.61
11	0.76	0.66
18	1.13	1.20
20	0.44	0.45
M	1.00	1.12
SD	0.60	0.63
SE	0.19	0.20

SSG	pre	post
9	0.34	0.70
8	0.49	1.17
1	0.55	0.23
3	0.60	0.56
4	0.93	0.51
7	1.75	1.44
5	1.65	0.61
10	1.85	0.79
2	1.41	0.67
6	0.43	0.25
M	1.00	0.69
SD	0.60	0.37
SE	0.19	0.12

MEF2A

CON	pre	post
25	0.15	0.52
23	1.09	1.31
27	0.49	0.12
22	0.33	0.09
32	0.88	0.54
28	0.90	1.50
26	0.15	0.05
24	0.56	0.18
31	4.45	1.95
M	1.00	0.69
SD	1.34	0.71
SE	0.45	0.24

CYC	pre	post
19	0.14	0.07
16	1.61	2.14
15	0.24	0.55
12	2.72	2.12
14	0.40	0.74
13	3.54	1.25
17	0.46	0.73
21	0.36	0.60
11	0.38	0.54
18	0.63	0.73
20	0.51	0.43
M	1.00	0.90
SD	1.19	0.69
SE	0.38	0.22

SSG	pre	post
9	0.39	1.58
8	0.59	2.11
1	1.63	0.58
3	0.63	0.65
4	0.56	0.22
7	1.81	2.83
5	1.10	0.60
10	2.77	1.88
2	0.35	0.13
6	0.17	0.06
M	1.00	1.06
SD	0.83	0.96
SE	0.26	0.30

Tfam

CON	pre	post
25	0.81	1.56
23	1.25	1.74
27	2.60	0.63
22	1.28	0.68
32	0.79	0.65
28	0.53	0.62
26	0.79	0.29
24	0.29	0.32
31	0.66	0.92
M	1.00	0.82
SD	0.68	0.51
SE	0.23	0.17

CYC	pre	post
19	0.43	0.45
16	2.07	1.34
15	0.36	1.19
12	3.51	2.74
14	1.81	0.40
13	0.93	0.76
17	0.22	0.77
21	0.47	0.24
11	0.51	0.33
18	0.64	0.46
20	0.06	0.24
M	1.00	0.81
SD	1.05	0.75
SE	0.33	0.24

SSG	pre	post
9	0.42	1.33
8	1.26	1.44
1	1.31	0.75
3	1.62	0.77
4	0.40	0.17
7	0.72	1.29
5	1.13	0.33
10	1.63	1.00
2	1.10	0.36
6	0.39	0.01
M	1.00	0.74
SD	0.48	0.51
SE	0.15	0.16

COXI

CON	pre	post
25	0.80	1.44
23	1.48	1.91
27	2.34	0.73
22	1.97	0.65
32	0.73	0.71
28	0.44	0.60
26	0.28	0.02
24	0.32	0.45
31	0.64	1.79
M	1.00	0.92
SD	0.75	0.64
SE	0.25	0.21

CYC	pre	post
19	0.39	0.54
16	2.64	1.87
15	0.37	1.41
12	2.01	2.12
14	2.38	0.88
13	0.95	1.82
17	0.56	1.77
21	0.63	0.56
11	0.54	1.30
18	0.22	0.74
20	0.31	0.27
M	1.00	1.20
SD	0.91	0.59
SE	0.27	0.18

SSG	pre	post
9	0.44	0.86
8	1.46	2.43
1	1.15	0.94
3	1.41	2.08
4	0.30	0.20
7	1.01	2.94
5	1.51	0.62
10	1.66	1.31
2	0.99	0.64
6	0.08	0.05
M	1.00	1.21
SD	0.55	0.97
SE	0.17	0.31

COXII

CON	pre	post
25	0.72	1.13
23	0.43	0.72
27	1.73	0.57
22	1.30	0.50
32	1.03	0.95
28	0.45	0.53
26	1.15	0.23
24	0.79	0.81
31	1.40	1.49
M	1.00	0.77
SD	0.44	0.38
SE	0.15	0.13

CYC	pre	post
19	0.37	0.46
16	1.36	1.29
15	0.39	0.96
12	2.05	2.36
14	2.55	0.88
13	1.09	1.38
17	0.48	1.03
21	0.70	0.48
11	0.64	1.67
18	1.00	1.42
20	0.36	0.36
M	1.00	1.12
SD	0.74	0.57
SE	0.22	0.17

SSG	pre	post
9	0.30	0.80
8	0.88	1.30
1	0.81	0.56
3	1.34	1.57
4	0.39	0.27
7	0.84	1.67
5	1.75	0.67
10	1.84	2.08
2	1.65	1.43
6	0.19	0.04
M	1.00	1.04
SD	0.62	0.67
SE	0.19	0.21

COXIII

CON	pre	post
25	0.34	0.94
23	0.87	0.86
27	1.08	0.39
22	0.92	0.27
32	2.79	0.81
28	0.38	0.37
26	1.03	0.48
24	0.87	0.69
31	0.73	0.80
M	1.00	0.62
SD	0.72	0.25
SE	0.24	0.08

CYC	pre	post
19	0.31	0.30
16	2.36	1.79
15	0.24	0.66
12	1.70	1.25
14	1.86	0.89
13	1.72	1.11
17	0.50	1.12
21	0.63	0.62
11	0.44	0.80
18	0.60	1.16
20	0.63	0.40
M	1.00	0.92
SD	0.78	0.41
SE	0.24	0.12

SSG	pre	post
9	0.46	0.99
8	1.19	2.32
1	0.70	0.45
3	1.07	1.00
4	0.54	0.26
7	1.16	2.52
5	1.44	1.27
10	2.03	0.94
2	1.22	0.59
6	0.21	0.18
M	1.00	1.05
SD	0.53	0.80
SE	0.17	0.25

COXIV

CON	pre	post
25	0.78	1.40
23	1.27	1.73
27	1.70	0.55
22	1.60	0.62
32	0.83	0.94
28	0.43	0.48
26	0.33	0.25
24	0.89	0.61
31	1.17	1.46
M	1.00	0.90
SD	0.48	0.52
SE	0.16	0.17

CYC	pre	post
19	0.40	0.59
16	2.76	1.87
15	0.38	1.26
12	2.07	2.89
14	2.23	0.85
13	1.18	1.57
17	0.64	1.57
21	0.61	0.51
11	0.16	0.65
18	0.23	0.53
20	0.33	0.38
M	1.00	1.15
SD	0.95	0.77
SE	0.29	0.23

SSG	pre	post
9	0.43	1.07
8	1.22	2.14
1	1.01	0.79
3	1.52	1.81
4	0.37	0.23
7	0.89	1.89
5	1.78	0.55
10	1.99	1.52
2	0.57	0.43
6	0.22	0.10
M	1.00	1.05
SD	0.62	0.74
SE	0.19	0.23

COXV

CON	pre	post
25	0.82	1.35
23	1.35	1.59
27	1.59	0.51
22	1.06	0.48
32	0.84	0.86
28	0.41	0.57
26	1.03	0.58
24	0.87	0.67
31	1.04	1.16
M	1.00	0.86
SD	0.34	0.41
SE	0.11	0.14

CYC	pre	post
19	0.44	0.49
16	2.69	1.94
15	0.29	0.79
12	1.85	1.28
14	1.65	0.78
13	1.55	0.93
17	0.66	1.11
21	0.59	0.47
11	0.38	0.66
18	0.49	0.83
20	0.41	0.40
M	1.00	0.88
SD	0.82	0.44
SE	0.25	0.13

SSG	pre	post
9	0.62	1.31
8	1.42	1.98
1	0.67	0.46
3	1.24	1.03
4	0.50	0.28
7	1.04	2.40
5	1.39	0.87
10	1.84	1.24
2	0.93	0.47
6	0.34	0.30
M	1.00	1.03
SD	0.47	0.72
SE	0.15	0.23

SIRT1

CON	pre	post
25	0.62	0.62
23	1.18	0.61
27	0.58	0.41
22	0.15	0.23
32	0.95	0.65
28	0.86	1.37
26	1.89	1.80
24	1.58	4.37
31	1.18	1.09
M	1.00	1.24
SD	0.53	1.27
SE	0.18	0.42

CYC	pre	post
19	0.39	0.40
16	0.37	0.38
15	2.26	0.51
12	0.32	0.22
14	0.83	0.98
13	0.89	1.30
17	0.21	1.02
21	1.16	0.96
11	1.90	1.53
18	1.52	1.95
20	1.14	1.16
M	1.00	0.95
SD	0.71	0.56
SE	0.22	0.17

SSG	pre	post
9	0.90	0.98
8	0.29	0.48
1	0.52	1.47
3	0.36	0.69
4	0.86	0.57
7	2.72	2.56
5	0.58	0.69
10	0.36	0.47
2	2.33	2.19
6	1.08	0.32
M	1.00	1.04
SD	0.85	0.78
SE	0.27	0.25

p53

CON	pre	post
25	0.98	1.03
23	1.17	0.94
27	1.09	1.06
22	1.61	0.95
32	1.29	1.27
28	1.01	1.26
26	0.19	0.18
24	0.62	1.81
31	1.04	1.33
M	1.00	1.09
SD	0.40	0.44
SE	0.13	0.15

CYC	pre	post
19	1.13	1.32
16	1.06	0.96
15	1.12	1.40
12	1.14	0.72
14	1.66	2.09
13	0.37	0.89
17	1.28	1.00
21	1.28	1.08
11	0.22	0.26
18	0.25	0.13
20	1.48	1.67
M	1.00	1.05
SD	0.49	0.56
SE	0.15	0.17

SSG	pre	post
9	1.44	1.26
8	1.25	1.16
1	1.19	1.15
3	1.07	1.11
4	1.21	1.30
7	0.81	2.32
5	1.61	1.49
10	0.64	1.41
2	0.12	0.30
6	0.65	1.03
M	1.00	1.25
SD	0.44	0.50
SE	0.14	0.16

AKT

CON	pre	post
25	0.33	1.32
23	1.03	0.64
27	0.86	0.55
22	0.20	0.13
32	2.38	0.55
28	0.28	0.35
26	1.97	1.01
24	1.11	0.66
31	0.85	1.36
M	1.00	0.73
SD	0.75	0.42
SE	0.25	0.14

CYC	pre	post
19	0.47	0.95
16	1.20	1.12
15	0.21	0.40
12	0.48	0.30
14	2.47	0.75
13	0.84	1.61
17	0.23	0.68
21	0.56	0.40
11	1.29	2.43
18	2.24	1.73
20	1.01	1.18
M	1.00	1.05
SD	0.80	0.69
SE	0.24	0.21

SSG	pre	post
9	0.63	1.30
8	0.72	1.13
1	0.36	0.20
3	0.12	0.06
4	4.19	1.26
7	0.62	2.92
5	0.32	0.18
10	0.14	0.12
2	2.19	2.45
6	0.71	0.45
M	1.00	1.01
SD	1.26	1.01
SE	0.40	0.32

PGC-1α

CON	pre	post
25	1.00	1.10
23	0.97	1.08
27	0.98	0.62
22	0.32	0.53
32	1.09	1.73
28	1.97	1.59
26	0.66	0.86
24	0.72	0.65
31	1.29	0.59
M	1.00	0.97
SD	0.46	0.44
SE	0.15	0.15

CYC	pre	post
BRJO	1.18	0.94
DALI	0.99	1.79
PAJE	0.61	0.70
DANE	0.82	0.61
PEIV	1.00	1.11
JAHO	1.24	0.76
RABL	1.15	1.31
ROPO	1.22	1.07
CHCO	1.04	0.47
BRLE	0.71	0.26
ADYA	1.05	1.30
M	1.00	0.94
SD	0.22	0.44
SE	0.07	0.13

SSG	pre	post
9	1.63	1.28
8	0.95	1.96
1	0.88	0.87
3	1.29	1.94
4	0.92	0.61
7	1.23	1.11
5	1.05	1.17
10	1.23	0.75
2	0.54	0.50
6	0.28	1.91
M	1.00	1.21
SD	0.39	0.56
SE	0.12	0.18

GLUT4

CON	pre	post
25	1.09	0.98
23	1.05	0.89
27	0.63	0.71
22	0.59	0.69
32	0.75	0.48
28	0.40	0.72
26	2.37	1.85
24	1.72	1.37
31	0.39	0.55
M	1.00	0.91
SD	0.66	0.44
SE	0.22	0.15

CYC	pre	post
19	0.85	0.90
16	0.94	0.89
15	0.68	0.57
12	0.45	0.45
14	0.74	0.69
13	0.62	0.62
17	0.97	0.55
21	0.72	0.62
11	1.76	1.72
18	1.61	1.26
20	1.67	1.87
M	1.00	0.92
SD	0.43	0.39
SE	0.13	0.12

SSG	pre	post
9	1.15	1.12
8	1.23	1.34
1	0.75	0.63
3	1.16	1.06
4	0.62	0.66
7	0.82	1.29
5	0.84	0.91
10	0.52	0.71
2	1.50	1.50
6	1.40	3.99
M	1.00	1.32
SD	0.33	0.99
SE	0.11	0.31
